# Germline mutation of *MDM4*, a major p53 regulator, in a familial syndrome of defective telomere maintenance

**DOI:** 10.1126/sciadv.aay3511

**Published:** 2020-04-10

**Authors:** Eléonore Toufektchan, Vincent Lejour, Romane Durand, Neelam Giri, Irena Draskovic, Boris Bardot, Pierre Laplante, Sara Jaber, Blanche P. Alter, José-Arturo Londono-Vallejo, Sharon A. Savage, Franck Toledo

**Affiliations:** 1 Genetics of Tumor Suppression, Institut Curie, Paris, France.; 2 CNRS UMR 3244, Paris, France.; 3 Sorbonne Université, Paris, France.; 4 PSL Research University, Paris, France.; 5 Clinical Genetics Branch, Division of Cancer Epidemiology and Genetics, National Cancer Institute, Rockville, MD, USA.; 6 Telomeres and Cancer, Institut Curie, Paris, France.

## Abstract

Dyskeratosis congenita is a cancer-prone inherited bone marrow failure syndrome caused by telomere dysfunction. A mouse model recently suggested that p53 regulates telomere metabolism, but the clinical relevance of this finding remained uncertain. Here, a germline missense mutation of *MDM4*, a negative regulator of p53, was found in a family with features suggestive of dyskeratosis congenita, e.g., bone marrow hypocellularity, short telomeres, tongue squamous cell carcinoma, and acute myeloid leukemia. Using a mouse model, we show that this mutation (p.T454M) leads to increased p53 activity, decreased telomere length, and bone marrow failure. Variations in p53 activity markedly altered the phenotype of *Mdm4* mutant mice, suggesting an explanation for the variable expressivity of disease symptoms in the family. Our data indicate that a germline activation of the p53 pathway may cause telomere dysfunction and point to polymorphisms affecting this pathway as potential genetic modifiers of telomere biology and bone marrow function.

## Teaser

We demonstrate a direct link between germline p53 activation and telomere dysfunction.

## INTRODUCTION

*TP53* is the gene most frequently mutated in human tumors (*1*), and germ line–inactivating p53 mutations cause the Li-Fraumeni syndrome of cancer predisposition (*2*). In addition, accelerated tumorigenesis has been associated with polymorphisms increasing the expression of MDM2 or MDM4, the essential p53 inhibitors (*3*, *4*). Alterations of the p53/MDM2/MDM4 regulatory node are, thus, mainly known to promote cancer. Unexpectedly, however, we recently found that mice expressing p53^Δ31^, a hyperactive mutant p53 lacking its C terminus, recapitulated the complete phenotype of patients with dyskeratosis congenita (DC) (*5*).

DC is a telomere biology disorder characterized by the mucocutaneous triad of abnormal skin pigmentation, nail dystrophy, and oral leukoplakia; patients are also at very high risk of bone marrow failure, pulmonary fibrosis, and cancer, especially head and neck squamous cell carcinoma (HNSCC) and acute myeloid leukemia (AML) (*6*). Patients with DC are known to exhibit disease diversity in terms of age of onset, symptoms, and severity due to the mode of inheritance and causative gene (*7*, *8*). DC is caused by germline mutations in genes encoding key components of telomere biology: the telomerase holoenzyme (*DKC1*, *TERC*, *TERT*, *NOP10*, and *NHP2*), the shelterin telomere protection complex (*ACD*, *TINF2*, and *POT1*), telomere capping proteins (*CTC1* and *STN1*), and other proteins interacting with these cellular processes (*RTEL1*, *NAF1*, *WRAP53*, and *PARN*) (*6*). Twenty to 30% of affected individuals remain unexplained at the molecular level.

Our finding that *p53*^Δ*31/*Δ*31*^ mice were remarkable models of DC was initially unexpected for two reasons. First, an increased p53 activity was not expected to cause telomere dysfunction, given the well-accepted notion that p53 acts as “the guardian of the genome”. However, p53 is now known to down-regulate the expression of many genes involved in genome maintenance (*5*, *9*, *10*), and this might actually contribute to its toolkit to prevent tumor formation (*11*). Second, telomere biology diseases are usually difficult to model in mice because of differences in telomere length and telomerase expression between mice and humans. Mice that lack telomerase exhibited short telomeres only after three or four generations (G3/G4) of intracrosses (*12*, *13*). However, mice with a telomerase haploinsufficiency and a deficient shelterin complex exhibited telomere dysfunction and DC features in a single generation (G1) (*14*). Because DC features were observed in G1 *p53*^Δ*31/*Δ*31*^ mice, we supposed that p53 might exert pleiotropic effects on telomere maintenance. Consistent with this, we found that murine p53 down-regulates several genes implicated in telomere biology (*5*, *9*). Because some of these genes were also down-regulated by p53 in human cells (*5*, *9*), our data suggested that an activating p53 mutation might cause features of DC in humans. However, this conclusion remained speculative in the absence of any clinical evidence.

Here, we report the identification of a germline missense mutation in *MDM4*, encoding an essential and specific negative regulator of p53, in a family presenting some DC-like phenotypic traits. We used a mouse model to demonstrate that this mutation leads to p53 activation, short telomeres, and bone marrow failure. Together, our results provide compelling evidence that a germline mutation affecting a specific p53 regulator may cause DC-like features in both humans and mice.

## RESULTS

### A *MDM4* mutation in a family with DC-like features

**Fig. 1:**
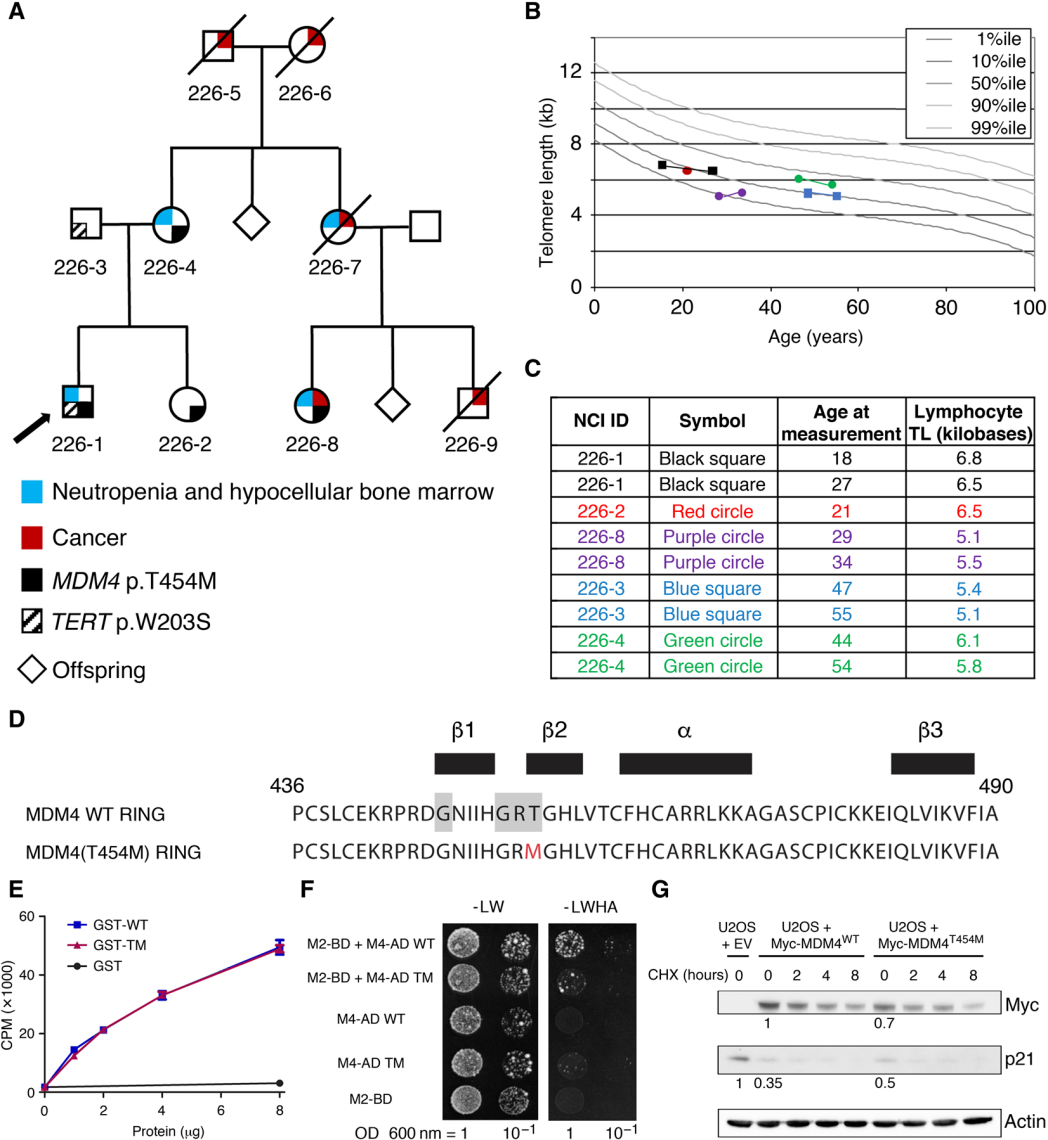
A germline *MDM4* p.T454M mutation identified in a family with bone marrow hypocellularity and short telomeres. (**A**) Pedigree of family NCI-226. Arrow indicates proband. Cancer histories include oral squamous cell carcinoma for 226-8 at age 27 years and for 226-9 at age 42 years, and melanoma at 51 years and AML at 52 years for 226-7 (see table S1 for further details). 226-5 had lung cancer at age 69 years. 226-6 had non-Hodgkin lymphoma at age 91 years. In addition, four siblings of 226-6 had cancer: one with breast, two with lung, and one with ovary or uterus (not specified). Sequencing of 226-5, 226-6, 226-7, and 226-9 was not possible because of lack of available DNA. (**B** and **C**) Lymphocyte telomere lengths (TL) of study participants. Total lymphocyte telomere lengths are shown and were measured by flow cytometry with in situ hybridization. (B) Graphical depiction of telomere length in relation to age. Four individuals had telomeres measured twice. Legend is in (C). Percentiles (%ile) are based on 400 healthy individuals (*50*). (C) Age at measurement(s) and telomere length in kilobases. (**D**) Sequence of the MDM4 RING domain (residues 436 to 490) with secondary structure residues indicated (black boxes). The P-loop motif is highlighted in gray, and the mutated residue in red. (**E**) The mutant RING domain retains ATP-binding capacity. Wild-type (WT) and mutant (TM) glutathione *S*-transferase (GST)–RING proteins, or GST alone, were incubated with 10 nM ATP and 5 μCi ATP-γ^32^P for 10 min at room temperature, filtered through nitrocellulose, and counted by liquid scintillation CPM, counts per minute. Results from two independent experiments. (**F**) The mutant MDM4 RING domain has an altered capacity to dimerize with the MDM2 RING. Two-hybrid assays were carried out as described (*47*). -LW, minus leucine and tryptophan; -LWHA, minus leucine, tryptophan, histidine and adenine; OD, optical density. Growth on the -LWHA medium indicates protein interaction, readily observed between MDM2 (M2-BD) and WT MDM4 (M4-AD WT) but faintly visible between MDM2 and MDM4^T454M^ (M4-AD TM). (**G**) Impact of the mutation in transfected human cells. U2OS cells were transfected with an empty vector (EV) or an expression plasmid encoding a Myc-tagged MDM4 (WT or T454M) protein and then treated or not with cycloheximide (CHX) to inhibit protein synthesis, and protein extracts were immunoblotted with antibodies against Myc, p21, or actin. Bands were normalized to actin, and a value of 1 was assigned to cells transfected with the WT MDM4 expression plasmid (for Myc) or with the empty vector (for p21).

Family NCI-226 first enrolled in the National Cancer Institute (NCI) inherited bone marrow failure syndrome (IBMFS) cohort in 2008 (Fig. 1A and table S1). At the time, the proband (226-1) was 17 years of age and had a history of neutropenia, bone marrow hypocellularity, vague gastrointestinal symptoms, and chronic pain. His mother (226-4) also had intermittent neutropenia and a hypocellular bone marrow. Notably, his maternal aunt (226-7) had a history of melanoma and died at age 52 because of AML. The maternal aunt’s daughter (proband’s cousin, 226-8) had HNSCC at age 27 years, intermittent neutropenia, and bone marrow hypocellularity, while her son (proband’s cousin, 226-9) was diagnosed with metastatic HNSCC at 42 years of age. The proband’s father (226-3) was healthy with the exception of hemochromatosis. An IBMFS was suspected on the basis of the family history of cancer and neutropenia. Chromosome breakage for Fanconi anemia was normal, while lymphocyte telomeres were between the 1st and 10th percentiles in the proband and maternal cousin (226-8) (Fig. 1, B and C). The proband was tested for mutations in known DC-causing genes, and a *TERT* variant (p.W203S) was identified. Unexpectedly, however, the variant was found to be inherited from his father. *TERT* p.W203S is not present in gnomAD, but it is predicted to be tolerated by MetaSVM (*15*).

Since the *TERT* variant did not track with disease inheritance, whole-exome sequencing (WES) was performed to search for a causal gene. The whole-exome data were filtered by maternal autosomal inheritance and revealed three genes with heterozygous missense mutations potentially deleterious according to bioinformatics predictions: *MDM4*, *KRT76*, and *REM1* (table S2). Given the limited knowledge of the function of *KRT76* and *REM1*, and our prior knowledge of a DC-like phenotype in *p53*^Δ*31/*Δ*31*^ mice, we chose to focus on the mutation affecting *MDM4* because it encodes a major negative regulator of p53. Although the T454M mutation does not affect the p53 interaction domain of MDM4, it might affect p53 regulation because it affects the MDM4 RING domain: Residue 454 is both part of a P-loop motif thought to confer adenosine triphosphate (ATP)–binding capacity (*16*) and part of a β strand important for MDM2-MDM4 heterodimerization (Fig. 1D) (*17*). The mutant RING domain had fully retained its capacity to bind ATP specifically (Fig. 1E and fig. S1A) but exhibited an altered capacity to interact with the MDM2 RING domain in a yeast two-hybrid assay (Fig. 1F). We next used transfection experiments to evaluate the consequences of this mutation on the full-length protein in human cells. We transfected U2OS cells—known to have a functional but attenuated p53 pathway due to MDM2 overexpression (*18*)—with either an empty vector or an expression plasmid encoding a Myc-tagged MDM4^WT^ or MDM4^T454M^ protein. Compared with cells transfected with the empty vector, cells transfected with a MDM4^WT^ or a MDM4^T454M^ expression plasmid exhibited decreased p21 levels, indicating MDM4-mediated p53 inhibition in both cases (Fig. 1G). However, the decrease in p21 levels was less pronounced in cells expressing MDM4^T454M^ than in cells expressing MDM4^WT^ (Fig. 1G) despite similar transfection efficiencies (fig. S1B). The lower expression levels of the MDM4^T454M^ protein likely contributed to its decreased capacity to inhibit p53 (Fig. 1G). In this experimental setting, the treatment with cycloheximide did not reveal any significant difference in stability between the mutant and wild-type (WT) MDM4 proteins (Fig. 1G and quantification in fig. S1C), raising the possibility that the observed lower MDM4^T454M^ protein levels might result from differences in mRNA translation efficiency. Together, these preliminary results argued for an impact of the mutation on MDM4 function, leading to p53 activation.

### Targeting of an Mdm4^T454M^ mutation in the mouse

**Fig. 2:**
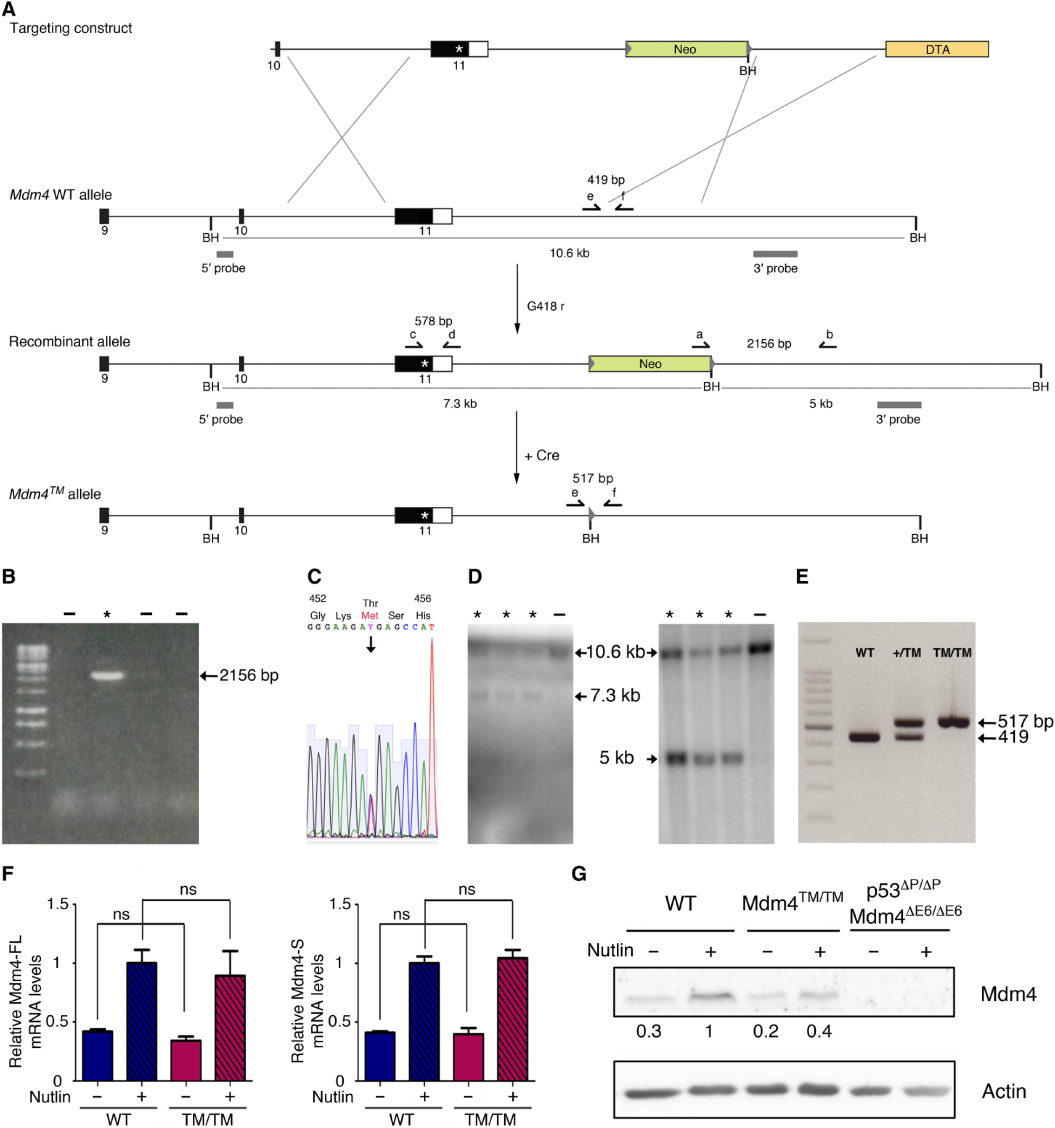
Generation of a mouse model with a targeted Mdm4^T454M^ mutation. (**A**) Targeting strategy. Homologous recombination in ES cells was used to target the T454M mutation at the *Mdm4* locus. For the *Mdm4* WT allele, exons 9 to 11 are shown [black boxes, coding sequences; white box, 3′ untranslated region (3′UTR)] and Bam HI (BH) restriction sites. Above, the targeting construct contains the following: (i) a 2.9-kb-long 5′ homology region encompassing exon 10, intron 10, and exon 11 sequences upstream the mutation; (ii) the mutation (asterisk) within exon 11; (iii) a 2.6-kb-long fragment encompassing the 3′ end of the gene and sequences immediately downstream; (iv) a *neomycin* selection gene (Neo) flanked by loxP sequences (gray arrowheads) and an additional BH site; (v) a 2.1-kb-long 3′ homology region containing sequences downstream *Mdm4*; and (vi) the *Diphtheria toxin* a gene (DTA) for targeting enrichment. (**B** to **D**) screening of G418-resistant ES clones as described in (A), with asterisks (*) indicating positive recombinants: (B) PCR with primers a and b; (C) sequencing after PCR with primers c and d: the sequence for codons 452 to 456 demonstrates heterozygosity at codon 454; (D) Southern blot of Bam HI–digested DNA with the 5′ (left) or 3′ (right) probe. (**E**) Examples of fibroblast genotyping by PCR with primers e and f. (**F**) The Mdm4^T454M^ mutation does not alter Mdm4 mRNA levels. Mdm4-FL (left) and Mdm4-S (right) mRNAs were extracted from WT and *Mdm4^TM/TM^* MEFs before or after treatment for 24 hours with 10 μM Nutlin, quantified using real-time PCR, and normalized to control mRNAs, and then the value in Nutlin-treated WT MEFs was assigned a value of 1. Results from five independent experiments and >4 MEFs per genotype. ns, not significant in a Student’s *t* test. (**G**) Decreased Mdm4 protein levels in *Mdm4^TM/TM^* MEFs. Protein extracts, prepared from MEFs treated as in (F), were immunoblotted with antibodies against Mdm4 or actin. Bands were normalized to actin, and then the values in Nutlin-treated WT cells were assigned a value of 1. *p53*^Δ*P/*Δ*P*^ *Mdm4*^Δ*E6/*Δ*E6*^ MEFs do not express a full-length Mdm4 protein (*20*): They were loaded to unambiguously identify the Mdm4(-FL) band in the other lanes.

The MDM4 RING domain is remarkably conserved throughout evolution, e.g., with 91% identity between the RING domains of human MDM4 and mouse Mdm4 (*19*). Thus, we decided to create a mouse model to precisely evaluate the physiological impact of the human mutation. We used homologous recombination in embryonic stem (ES) cells to target the p.T454M mutation at the *Mdm4* locus (Fig. 2A). Targeted recombinants were identified by long-range polymerase chain reaction (PCR) (Fig. 2B), confirmed by DNA sequencing (Fig. 2C), and the structure of the recombinant allele was further analyzed by Southern blots with probes located 5′ and 3′ of the targeted mutation (Fig. 2D). Recombinant ES clones were then microinjected into blastocysts to generate chimeric mice, and chimeras were mated with PGK-Cre mice to excise the Neo gene. PCR was used to verify transmission through the germ line of the *Mdm4^T454M^* (noted below *Mdm4^TM^*) mutation and to genotype the mouse colony and mouse embryonic fibroblasts (MEFs) (Fig. 2E). We first isolated RNAs from *Mdm4^TM/TM^* MEFs and sequenced the entire Mdm4 coding sequence: The Mdm4^TM^ sequence was identical to the WT Mdm4 sequence except for the introduced missense mutation (not shown). Furthermore, like its human counterpart, the *Mdm4* gene encodes two major transcripts: Mdm4-FL, encoding the full-length oncoprotein that inhibits p53, and Mdm4-S, encoding a shorter, extremely unstable protein (*20*, *21*). We observed, in unstressed cells as well as in cells treated with Nutlin [a molecule that activates p53 by preventing Mdm2-p53 interactions (*22*) without altering Mdm4-p53 interactions (*23*, *24*)], that the Mdm4^TM^ mutation affected neither Mdm4-FL nor Mdm4-S mRNA levels (Fig. 2F). In Western blots, however, Mdm4-FL was the only detectable isoform, and it was expressed at lower levels in the mutant MEFs (Fig. 2G).

### *Mdm4^TM/TM^* fibroblasts exhibit increased p53 activity and short telomeres

**Fig. 3:**
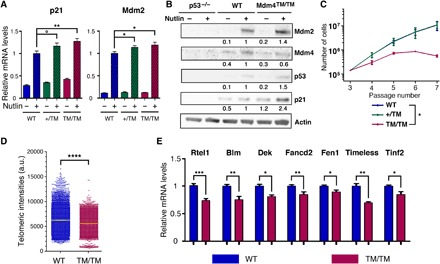
*Mdm4^TM/TM^* fibroblasts exhibit increased p53 activity and short telomeres. (**A**) Quantification of p21 and Mdm2 mRNAs extracted from WT, *Mdm4^+/TM^*, and *Mdm4^TM/TM^* MEFs, treated or not for 24 hours with 10 μM Nutlin. mRNA levels were quantified using real-time PCR and normalized to control mRNAs, and then the value in Nutlin-treated WT MEFs was assigned a value of 1. Results from 10 independent experiments. (**B**) Protein extracts, prepared from *p53^−/−^*, WT, and *Mdm4^TM/TM^* MEFs treated as in (A), were immunoblotted with antibodies against Mdm2, Mdm4, p53, p21, or actin. Bands were normalized to actin, and then the values in Nutlin-treated WT MEFs were assigned a value of 1. (**C**) Proliferation of MEFs in a 3T3 protocol. Each point is the average value of three independent MEFs. (**D**) Decreased telomere length in *Mdm4^TM/TM^* MEFs, as measured by quantitative FISH with a telomeric probe. Results from two MEFs per genotype, and 68 to 75 metaphases per MEF [means + 95% confidence interval (CI) are shown in yellow]. a.u., arbitrary units. (**E**) Telomere-related genes down-regulated in *Mdm4^TM/TM^* MEFs. mRNAs were extracted from unstressed WT and *Mdm4^TM//TM^* MEFs, quantified using real-time PCR, and normalized to control mRNAs, and the value in WT MEFs was assigned a value of 1. Results from >3 independent experiments and two MEFs per genotype. In relevant panels: °*P* = 0.08, *P < 0.05, **P < 0.01, ***P < 0.001, and ****P < 0.0001 by Student’s *t* (A, C at passage 7, and E) or Mann-Whitney (D) statistical tests.

*Mdm4^TM/TM^* MEFs contained higher mRNA levels for the p53 targets *p21(Cdkn1a)* and *Mdm2*, indicating increased p53 activity (Fig. 3A). Consistent with this, *Mdm4^TM/TM^* MEFs exhibited increased p21 and Mdm2 protein levels (Fig. 3B and fig. S2). Moreover, *Mdm4^TM/TM^* MEFs prematurely ceased to proliferate when submitted to a 3T3 protocol (Fig. 3C), which also suggests an increased p53 activity. The mean telomere length was decreased by 11% in *Mdm4^TM/TM^* MEFs, and a subset of very short telomeres was observed in these cells, hence demonstrating a direct link between the Mdm4^TM^ mutation, p53 activation, and altered telomere biology (Fig. 3D). In *p53*^Δ*31/*Δ*31*^ MEFs, subtle but significant decreases in expression were previously observed for several genes involved in telomere biology, and in particular, small variations in *Rtel1* gene expression were found to have marked effects on the survival of *p53*^Δ*31/*Δ*31*^ mice (*5*, *9*). Similarly, *Mdm4^TM/TM^* MEFs exhibited subtle but significant decreases in expression for *Rtel1* and several other genes contributing to telomere biology (Fig. 3E). We previously showed that p53 activation correlates with an increased binding of the E2F4 repressor at the *Rtel1* promoter (*9*). Hence, the decreased Rtel1 mRNA levels in *Mdm4^TM/TM^* MEFs most likely resulted from increased p53 signaling. Consistent with this, a further increase in p53 activity, induced by Nutlin, led to further decreases in Rtel1 mRNA and protein levels, in both WT and *Mdm4^TM/TM^* cells (fig. S3A). Recently, in apparent contradiction with our finding that p53 activation can cause telomere shortening (*5*), p53 was proposed to prevent telomere DNA degradation by inducing subtelomeric transcripts, including telomere repeat-containing RNA (TERRA) (*25*, *26*), which suggested a complex, possibly context-dependent impact of p53 on telomeres (*27*). This led us to compare TERRA transcripts in WT and *Mdm4^TM/TM^* cells. Consistent with an earlier report (*26*), p53 activation led to increased TERRA at the mouse Xq subtelomeric region in WT cells (fig. S3B). However, *Mdm4^TM/TM^* cells failed to induce TERRA in response to stress (fig. S3B). Together, our data suggest that the telomere shortening observed in *Mdm4^TM/TM^* cells results from a p53-dependent decrease in expression of several telomere-related genes and, notably, *Rtel1*, a gene mutated in several families with DC (*6*). In addition, although evidence that altered TERRA levels can cause DC is currently lacking, we cannot exclude that an altered regulation of TERRA expression might contribute to telomere defects in *Mdm4^TM/TM^* cells.

### The perinatal death of *Mdm4^TM/TM^* mice is rescued by p53 loss

**Fig. 4:**
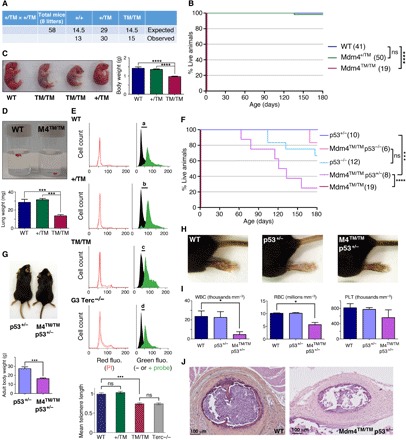
*Mdm4^TM/TM^* mice die perinatally but can be rescued by p53 loss or haploinsufficiency. (**A**) Mendelian distribution of the offspring from 8 *Mdm4^+/TM^* intercrosses. (**B**) *Mdm4^TM/TM^* mice die at birth. Cohort sizes are in parentheses. (**C**) *Mdm4^TM/TM^* neonates are smaller than their littermates and appear hypoxic. (**D**) Lungs from *Mdm4^TM/TM^* P0 pups are hypoplastic and sink in phosphate-buffered saline owing to a lack of air inflation. (**E**) Flow-FISH analysis of P0 lung cells with a telomere-specific peptide nucleic acid (PNA) probe. Top: Representative results from a WT, a *Mdm4^+/TM^*, a *Mdm4^TM/TM^*, and a G3 *Terc*^−/−^ mouse are shown. Right: Green fluorescence (fluo.) with black histograms for cells without the probe (measuring cellular autofluorescence) and green histograms for cells with the probe. The shift in fluorescence intensity is smaller in *Mdm4^TM/TM^* and *Terc*^−/−^ cells (c or d < a or b), indicating reduced telomere length. Left: Propidium iodide (PI) fluorescence histograms are superposed for cells with or without the probe. Below: Statistical analysis of green fluorescence shifts (see Materials and Methods). Means + 95% CI are shown; data are from two to three mice and >3800 cells per genotype. (**F**) Impact of decreased p53 activity on *Mdm4^TM/TM^* animals. Cohort sizes are in parentheses. (**G**) Examples of littermates with indicated genotypes. (**H**) Hind legs of mice with indicated genotypes. (**I**) *Mdm4^TM/TM^ p53^+/−^* mice exhibit abnormal hemograms. Counts for white blood cells (WBC), red blood cells (RBC), and platelets (PLT) for age-matched (120 days old) animals are shown. (**J**) Hematoxylin and eosin staining of sternum sections from WT and *Mdm4^TM/TM^ p53^+/−^* mice. In relevant panels: ns, not significant; *P < 0.05, ***P < 0.001, and ****P < 0.0001 by Mantel-Cox (B and F), Student’s *t* (C, D, G, and I), or Mann-Whitney (E) statistical tests. Photo credits: E.T. and R.D., Institut Curie (C, G, and H); R.D., Institut Curie (D).

*Mdm4^TM/TM^* mice were born in Mendelian proportions from *Mdm4^+/TM^* intercrosses (Fig. 4A) but were smaller than their littermates and died within 0 to 30 min after birth, with signs of severe respiratory distress (Fig. 4, B and C). Consistent with this, *Mdm4^TM/TM^* pups at postnatal day 0 (P0) appeared hypoxic (Fig. 4C), and their lungs were very small and dysfunctional (Fig. 4D). Thus, *Mdm4^TM/TM^* pups most likely died from neonatal respiratory failure. Tissues from *Mdm4^TM/TM^* pups exhibited increased p21 mRNA levels, suggesting an increase in p53 activity in these animals (fig. S4). We next used flow–FISH (fluorescence in situ hybridization) with a telomere-specific probe to evaluate the impact of the mutation on telomere length in vivo. Lung cells from *Mdm4^TM/TM^* pups (and control G3 *Terc*^−/−^ mice) exhibited a 25% decrease in mean telomere length compared with cells from WT or *Mdm4^+/TM^* littermates, indicating altered telomere biology in G1 homozygous mutants (Fig. 4E). Notably, p53 loss or haploinsufficiency rescued the perinatal lethality of *Mdm4^TM/TM^* pups, illustrating that the premature death of *Mdm4^TM/TM^* mice likely resulted from increased p53 activity (Fig. 4F). However, *p53^−/−^* and *Mdm4^TM/TM^ p53^−/−^* mice exhibited similar survival curves, with a fraction of the mice (respectively 4 of 12 and 1 of 6) succumbing to thymic lymphoma in less than 180 days. In contrast, after 180 days, all the *p53^+/−^* mice remained alive, whereas most *Mdm4^TM/TM^ p53^+/−^* mice had died. *Mdm4^TM/TM^ p53^+/−^* mice were smaller than their littermates (Fig. 4G) and exhibited hyperpigmentation of the footpads (Fig. 4H), and 120-day-old *Mdm4^TM/TM^ p53^+/−^* mice exhibited abnormal hemograms (Fig. 4I). Furthermore, the *Mdm4^TM/TM^ p53^+/−^* mice that died 60 to 160 days after birth exhibited bone marrow hypocellularity (Fig. 4J), indicating bone marrow failure as the likely cause for their premature death.

### *Mdm4^+/TM^* mice are hypersensitive to increases in p53 activity

**Fig. 5:**
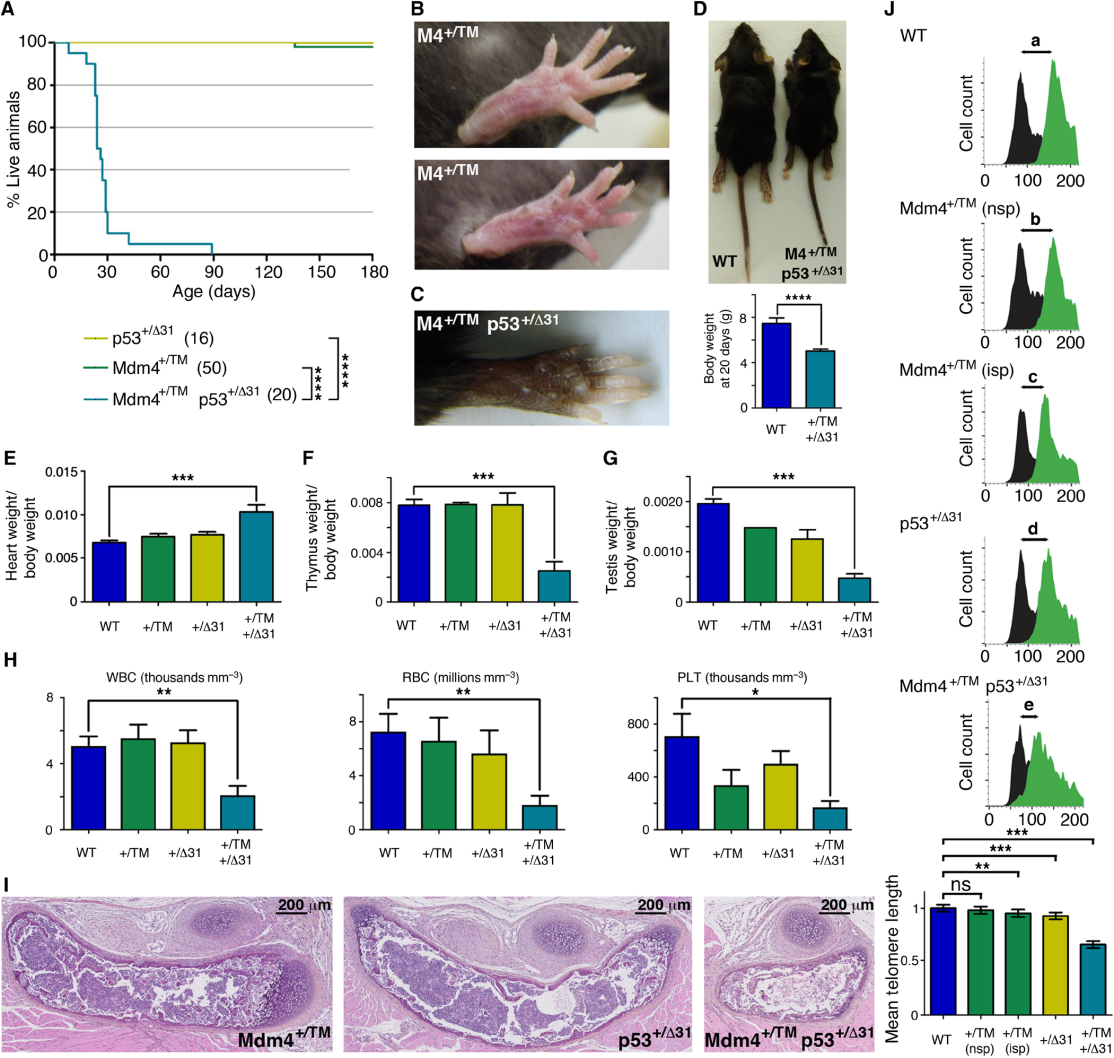
*Mdm4^+/TM^* mice are hypersensitive to increased p53 activity. (**A**) Impact of increased p53 activity on *Mdm4^+/TM^* animals. Cohort sizes are in parentheses. (**B**) Footpads from *Mdm4^+/TM^* mice appear normal (top) or exhibit a subtle increase in pigmentation (bottom). (**C**) *Mdm4^+/TM^* *p53*^*+/*Δ*31*^ mice exhibit strong skin hyperpigmentation. (**D**) *Mdm4^+/TM^* *p53*^*+/*Δ*31*^ mice are smaller than age-matched WT mice. (**E** to **G**) *Mdm4^+/TM^* *p53*^*+/*Δ*31*^ mice exhibit heart hypertrophy (E) as well as thymic (F) and testicular (G) hypoplasia. (**H**) *Mdm4^+/TM^* *p53*^*+/*Δ*31*^ mice exhibit abnormal hemograms. Counts for white blood cells, red blood cells, and platelets for five age-matched (P18) animals per genotype are shown. (**I**) Hematoxylin and eosin staining of sternum sections from mice of the indicated genotypes. (**J**) Flow-FISH analysis of P18 bone marrow cells with a telomere-specific PNA probe. Top: Representative results for a WT, a *Mdm4^+/TM^* with normal skin pigmentation (nsp), a *Mdm4^+/TM^* with increased footpad skin pigmentation (isp), a *p53*^*+/*Δ*31*^, and a *Mdm4^+/TM^* *p53*^*+/*Δ*31*^ mouse are shown; black histograms, cells without the probe; green histograms, cells with the probe. The smallest shift in fluorescence intensity (e) was observed with *Mdm4^+/TM^* *p53*^*+/*Δ*31*^ cells. Bottom: Statistical analysis of green fluorescence shifts. Means + 95% CI are shown; data are from >1500 cells per genotype. In relevant panels: ns, not significant; *P < 0.05, **P < 0.01, ***P < 0.001, and ****P < 0.0001 by Mantel-Cox (A), Student’s *t* (D and E to H), or Mann-Whitney (J) statistical tests. Photo credits: R.D. and P.L., Institut Curie (B); E.T. and R.D., Institut Curie (C and D).

Although *Mdm4^TM/TM^* MEFs and mice were useful to demonstrate that the Mdm4^T454M^ mutation leads to p53 activation and short telomeres, a detailed analysis of *Mdm4^+/TM^* mice appeared more relevant to model the NCI-226 family, in which all affected relatives were heterozygous carriers of the *MDM4^T454M^* mutation. Unlike *Mdm4^TM/TM^* mice, most *Mdm4^+/TM^* animals remained alive 6 months after birth and had no apparent phenotype, similarly to WT mice (Fig. 5A). This was consistent with our analyses in fibroblasts because *Mdm4^+/TM^* MEFs behaved like WT cells in a 3T3 proliferation assay (Fig. 3C). However, p53 target genes appeared to be transactivated slightly more efficiently in *Mdm4^+/TM^* than in WT cells (Fig. 3A), and 30% of *Mdm4^+/TM^* mice exhibited a slight hyperpigmentation of the footpads, suggesting a subtle increase in p53 activity (Fig. 5B). We reasoned that a further, subtle increase in p53 activity might affect the survival of *Mdm4^+/TM^* mice. We tested this hypothesis by mating *Mdm4^+/TM^* animals with *p53*^*+/*Δ*31*^ mice. *p53*^*+/*Δ*31*^ mice were previously found to exhibit a slight increase in p53 activity and to remain alive for over a year (*5*). Notably, unlike *Mdm4^+/TM^* or *p53*^*+/*Δ*31*^ heterozygous mice, *Mdm4^+/TM^* *p53*^*+/*Δ*31*^ compound heterozygotes died in less than 3 months (Fig. 5A) and exhibited many features associated with strong p53 activation. *Mdm4^+/TM^* *p53*^*+/*Δ*31*^ mice exhibited intense skin hyperpigmentation (Fig. 5C), were much smaller than their littermates (Fig. 5D), and exhibited heart hypertrophy (Fig. 5E) and thymic hypoplasia (Fig. 5F) and the males had testicular hypoplasia (Fig. 5G). Bone marrow failure was the likely cause for the premature death of *Mdm4^+/TM^* *p53*^*+/*Δ*31*^ mice, as indicated by abnormal hemograms of 18-day-old (P18) compound heterozygotes (Fig. 5H) and bone marrow hypocellularity in the sternum sections of moribund *Mdm4^+/TM^* *p53*^*+/*Δ*31*^ animals (Fig. 5I). We next used flow-FISH to analyze telomere length in the bone marrow cells of P18 WT, *Mdm4^+/TM^*, *p53*^*+/*Δ*31*^, and *Mdm4^+/TM^* *p53*^*+/*Δ*31*^ mice. We found no significant difference between telomere lengths in cells from five WT and three *Mdm4^+/TM^* mice with normal skin pigmentation, whereas cells from two *Mdm4^+/TM^* mice with increased skin pigmentation (or from *p53*^*+/*Δ*31*^ mice) exhibited marginal (5 to 7%) decreases in mean telomere length. Notably, in G1 *Mdm4^+/TM^* *p53*^*+/*Δ*31*^ cells, the average telomere length was decreased by 34% (Fig. 5J). Together, these results demonstrate that *Mdm4^+/TM^* mice are hypersensitive to subtle increases in p53 activity. Consistent with this, *Mdm4^+/TM^* *p53*^*+/*Δ*31*^ MEFs also exhibited increased p53 signaling and accelerated proliferation arrest in a 3T3 protocol (fig. S5). In sum, the comparison between *Mdm4^TM/TM^* and *Mdm4^TM/TM^ p53^+/−^* mice, or between *Mdm4^+/TM^* and *Mdm4^+/TM^* *p53*^*+/*Δ*31*^ animals, indicated that subtle variations in p53 signaling had marked effects on the phenotypic consequences of the Mdm4^T454M^ mutation (table S3).

### Polymorphisms that may affect p53 signaling differ among DC-like family members

**Fig. 6:**
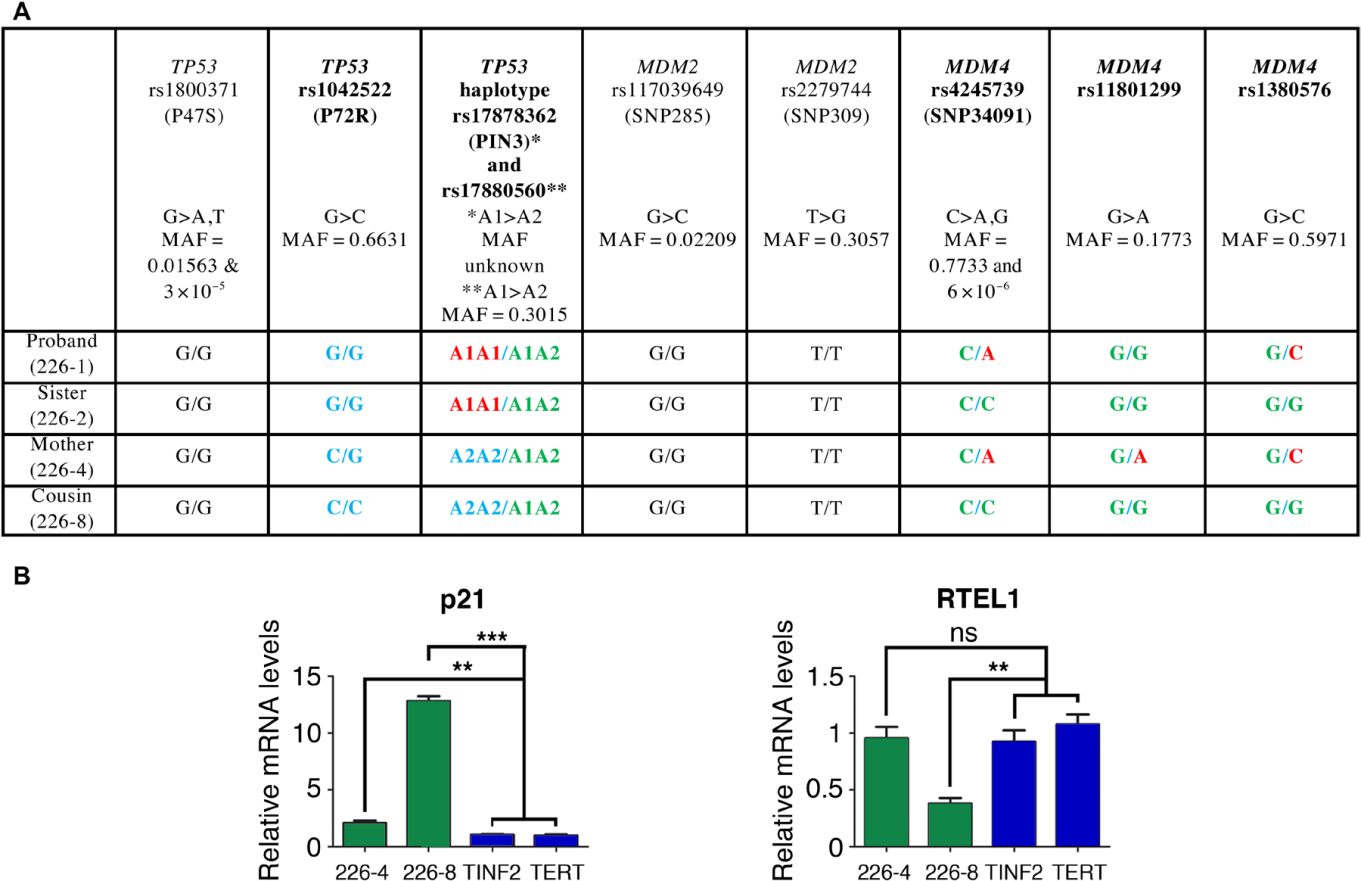
*TP53* or *MDM4* polymorphisms might alter the impact of the MDM4^T454M^ mutation among NCI-226 family members. (**A**) Genotyping of polymorphisms that may affect the p53 pathway. The SNPs rs1800371 and rs1042522 modify the p53 protein sequence (*28*,*29*), whereas rs17878362 and rs17880560 are singlets (A1) or doublets (A2) of G-rich sequences in noncoding regions of *TP53* that affect p53 expression (*30*). SNPs rs117039649 and rs2279744, in the *MDM2* promoter, affect MDM2 mRNA levels (*3*,*31*). Three SNPs are at the *MDM4* locus: rs4245739 in the 3′UTR region affects MDM4 protein levels (*4*), whereas rs11801299 and rs1380576 were associated with an increased risk of developing retinoblastoma (*32*), a cancer type with frequent MDM4 alterations (*51*). Polymorphisms that differ among family members are in bold, with the allele (or haplotype) associated with increased p53 activity in green (because it may synergize with the effects of the MDM4^T454M^ mutation). Alleles (or haplotypes) for which there is evidence of decreased p53 activity, or for which the effect is uncertain, are highlighted in red or blue, respectively. Please note that the clinical effects of the *TP53* rs1042522 SNP have recently been contested (*33*), so that all alleles for this SNP were labeled in blue. MAF, minor allele frequency reported for all gnomAD populations combined. https://gnomad.broadinstitute.org (*52*). (**B**) Comparative analysis of primary fibroblasts from family members 226-4 and 226-8. p21 and RTEL1 mRNAs, extracted from cells from relatives NCI 226-4 and NCI 226-8 or two unrelated patients with DC carrying a *TINF2* or a *TERT* mutation, were quantified using real-time PCR, normalized to control mRNAs, and then expressed relative to the mean values in *TINF2* and *TERT* mutant cells. ns, not significant, **P < 0.01 and ***P < 0.001 in a Student’s *t* test.

The carriers of the *MDM4^T454M^* mutation exhibited considerable heterogeneity in their phenotypes (Fig. 1 and table S1). The data from our mouse model suggested that variations in p53 activity might account for the variable expressivity and penetrance of clinical features among the NCI-226 *MDM4^+/T454M^* relatives. Hence, we analyzed nine known common polymorphisms reported to affect p53 activity and tumorigenesis (four at the *TP53* locus, two at the *MDM2* locus, and three at the *MDM4* locus) (*3*,*4*,*28*–*32*). Among the four *MDM4^+/T454M^* relatives, the proband (NCI-226-1) is more difficult to interpret because the potential contribution of the *TERT* p.W203S variant to his phenotype cannot be ruled out (even though it appears unlikely according to in silico predictions). The *MDM4* allele encoding the mutant protein (p.T454M) appears associated with the C allele of single-nucleotide polymorphism (SNP) rs4245739, the G allele of SNP rs11801299, and the G allele of SNP rs1380576 (Fig. 6A). These three *MDM4* variant alleles are associated with increased p53 activity (*4*,*32*) and might, thus, synergize with the *MDM4^T454M^* mutation in this family.

The proband’s affected cousin (226-8) exhibited a very early onset of disease, with lymphocyte telomere length within or below the first percentile of age-matched control participants and tongue squamous cell carcinoma at age 27 (Fig. 1 and table S1). The WT *MDM4* allele of 226-8 carried the rs4245739 C, the rs11801299 G, and the rs1380576 G variants associated with increased p53 activity. This suggests a potential disease-modifying effect of these *MDM4* SNPs. In contrast, the proband’s mother (226-4) was much less severely affected, with telomere length between the 10th and 50th percentiles (Fig. 1). Although we cannot rule out that disease anticipation might contribute to her milder phenotype, note that her WT MDM4 allele carried variants that might correlate with decreased p53 activity and could antagonize the *MDM4^T454M^* mutation (rs4245739 A, rs11801299 A, and rs1380576 C; Fig. 6A). Family members 226-4 and 226-8 shared the same genotypes for all the other tested variants, except for *TP53* rs1042522, a SNP first reported to affect apoptotic or cell cycle arrest responses (*28*), but with a clinical effect that now appears controversial (*33*). The proband’s sister (226-2), with a B cell deficiency and telomere lengths around the 10th percentile, also appeared less affected than 226-8. All the tested variants at the *MDM2* and *MDM4* loci were identical between 226-2 and 226-8. However, unlike 226-8, 226-2 exhibited a *TP53* allele with an A1A1 haplotype for variants rs17878362 and rs17880560 that might decrease p53 activity (*30*) and antagonize the effects of the *MDM4^T454M^* mutation (Fig. 6A).

We had primary fibroblasts available for two of these family members, 226-4 and 226-8, allowing us to directly assess the functional effect of the *MDM4^T454M^* variant in these cells. These fibroblasts were grown in parallel with primary fibroblasts from patients with DC carrying either a *TINF2^K280E^* mutation or a *TERT^P704S^* mutation, and mRNA levels for p21 and RTEL1 were quantified. In agreement with the notion that a *MDM4^T454M^* heterozygous mutation activates p53 signaling in NCI-226 family members, fibroblasts from both 226-4 and 226-8 exhibited increased p21 mRNA levels compared with *TINF2* or *TERT* mutant cells (Fig. 6B). However, cells from 226-4 only exhibited a 2-fold increase in p21 levels, whereas a 12-fold increase was observed for cells from 226-8, consistent with the notion that SNPs affecting the p53 pathway might counteract (for 226-4) or strengthen (for 226-8) the effect of the *MDM4^T454M^* mutation. Furthermore, we previously showed that RTEL1 mRNA levels are down-regulated upon p53 activation in human cells (*5*). RTEL1 mRNA levels appeared normal in cells from 226-4 but were markedly decreased in cells from 226-8, raising the possibility that a threshold in p53 activation might be required to affect RTEL1 expression (Fig. 6B).

## DISCUSSION

Although MDM4 is primarily known for its clinical relevance in cancer biology, our study shows that a germline missense *MDM4* mutation may cause features suggestive of DC. In humans, the *MDM4* (p.T454M) mutation was identified in this family with neutropenia, bone marrow hypocellularity, early-onset tongue SCC, AML, and telomeres between the 1st and 10th percentiles in the younger generation. In mice, the same *Mdm4* mutation notably correlated with increased p53 activity, short telomeres, and bone marrow failure. In both human transfected cells and MEFs, the mutant protein was expressed at lower levels than its WT counterpart, likely contributing to increased p53 activity. Together, these results demonstrate the importance of the MDM4/p53 regulatory axis on telomere biology and DC-like features in both species. Notably, *p53*^Δ*31/*Δ*31*^ mice were previously found to phenocopy DC (*5*), but whether this finding was relevant to human disease had remained controversial. When a mutation in *PARN* was found to cause DC (*34*), it first appeared consistent with the *p53*^Δ*31*^ mouse model because PARN, the polyadenylate-specific ribonuclease, had been proposed to regulate p53 mRNA stability (*35*). However, whether PARN regulates the stability of mRNAs is now contested (*36*). Rather, PARN would regulate the levels of over 200 microRNAs, of which only a few might repress p53 mRNA translation (*37*). Furthermore, PARN regulates TERC, the telomerase RNA component (*38*), and TERC overexpression increased telomere length in PARN-deficient cells (*39*). Thus, whether a germline mutation that specifically activates p53 can cause DC-like features remained to be demonstrated in humans, and our report provides compelling evidence for this, because unlike PARN, MDM4 is a very specific regulator of p53.

A germline antiterminating *MDM2* mutation was recently identified in a patient with a Werner-like syndrome of premature aging. Although multiple mechanisms might contribute to the clinical features in that report, a premature cellular senescence resulting from p53 hyperactivation was proposed to play a major role in his segmental progeroid phenotype (*40*). In that regard, our finding that increased p53 activity correlates with short telomeres appears relevant because telomere attrition is a primary hallmark of aging, well known to trigger cellular senescence (*41*). Furthermore, germline *TP53* frameshift mutations were recently reported in two patients diagnosed with pure red blood cell aplasia and hypogammaglobulinemia, resembling but not entirely consistent with Diamond Blackfan anemia (DBA) (*42*). In addition to the pure red cell aplasia diagnostic of DBA, those patients were found to exhibit relatively short telomeres (although not as short as telomeres from patients with DC), which may also seem consistent with our results. Our finding of an *MDM4* missense mutation in a DC-like family, together with recent reports linking an antiterminating *MDM2* mutation to a Werner-like phenotype and *TP53* frameshift mutations to DBA-like features, indicates that the clinical impact of germline mutations affecting the p53/MDM2/MDM4 regulatory network is just emerging. An inherited hyperactivation of the p53 pathway—via a germline *TP53*, *MDM2*, or *MDM4* mutation—may thus cause either DBA, Werner-like, or DC-like features, but additional work will be required to determine whether mutations in any of these three genes can cause any of these three syndromes. Likewise, several mouse models have implicated p53 deregulation in features of other developmental syndromes including the CHARGE, Treacher-Collins, Waardenburg, or DiGeorge syndrome (*43*), and it will be important to know whether germline mutations in *TP53*, *MDM2*, or *MDM4* may cause these additional syndromes in humans.

Heterozygous *Mdm4^+/TM^* mice appeared normal but were hypersensitive to variations in p53 activity, and, perhaps most notably, *Mdm4^+/TM^ p53*^*+/*Δ*31*^ compound heterozygous mice rapidly died from bone marrow failure. Thus, the p53^Δ31^ mutation acted as a strong genetic modifier of the Mdm4^TM^ mutation. It is tempting to speculate that similarly, among the NCI-226 family members heterozygous for the *MDM4^T454M^* allele, differences in the severity of phenotypic traits (e.g., lymphocyte telomere length and bone marrow cellularity) may result, in part, from modifiers affecting the p53 pathway and synergize or antagonize with the effects of the MDM4^T454M^ mutation. To search for potentially relevant modifiers, we looked at nine polymorphisms at the *TP53*, *MDM2*, and *MDM4* loci that were previously reported to affect p53 activity. Notably, we found that the family member most severely affected (226-8, the proband’s cousin) carried a *TP53* haplotype, as well as SNPs on the WT *MDM4* allele, that might synergize with the effects of the *MDM4^T454M^* mutation. Conversely, a *TP53* haplotype for the proband’s sister (226-2), or SNPs at the WT *MDM4* locus for the proband’s mother (226-4), might antagonize the impact of *MDM4^T454M^* allele. Consistent with this, primary fibroblasts from 226-4 and 226-8 exhibited increased p53 activity, but p53 activation was much stronger in cells from 226-8. Our data, thus, appear consistent with the existence of genetic modifiers at the *TP53* and *MDM4* loci that may affect DC-like phenotypic traits among family members carrying the *MDM4* (p.T454M) mutation. However, this remains speculative given the small number of individuals that could be analyzed. Furthermore, nonexonic variants affecting other genes might also contribute to DC-like traits (*44*). Last, the *TP53* and *MDM4* polymorphisms considered here were previously evaluated for their potential impact on tumorigenic processes, rather than DC-like traits such as telomere length or bone marrow hypocellularity. Our data suggest that polymorphisms at the *TP53* and *MDM4* (and possibly *MDM2*) loci should be evaluated for their potential impact on bone marrow function and telomere biology.

## MATERIALS AND METHODS

### Patient and family

The individuals in this study are participants in an Institutional Review Board–approved longitudinal cohort study at the NCI entitled “Etiologic Investigation of Cancer Susceptibility in Inherited Bone Marrow Failure Syndromes” (www.marrowfailure.cancer.gov, ClinicalTrials.gov NCT00027274) (*7*). Patients and their family members enrolled in 2008 and completed detailed family history and medical history questionnaires. Detailed medical record review and thorough clinical evaluations of the proband, his sister, parents, and maternal cousin were conducted at the National Institutes of Health (NIH) Clinical Center. Telomere length was measured by flow cytometry with in situ hybridization (flow-FISH) (*45*) in leukocytes of all patients and family members reported. DNA was extracted from whole blood using standard methods. DNA was not available from 226-7 or 226-9 (Fig. 1). Given the time frame of participant enrollment, Sanger sequencing of *DKC1*, *TINF2*, *TERT*, *TERC*, and *WRAP53* was performed first, followed by exome sequencing.

### Whole-exome sequencing

WES of blood-derived DNA for family NCI-226 was performed at the NCI’s Cancer Genomics Research Laboratory as previously described (*46*). Exome enrichment was performed with NimbleGen’s SeqCap EZ Human Exome Library v3.0 + UTR (Roche NimbleGen Inc., Madison, WI, USA), targeting 96 Mb of exonic sequence and the flanking untranslated regions (UTRs) on an Illumina HiSeq. Annotation of each exome variant locus was performed using a custom software pipeline. WES variants of interest were identified if they met the following criteria: heterozygous in the proband, his mother, and maternal cousin; nonsynonymous; had a minor allele frequency <0.1% in the Exome Aggregation Consortium databases; and occurred <5 times in our in house database of 4091 individuals. Variants of interest were validated to rule out false-positive findings using an Ion 316 chip on the Ion PGM Sequencer (Life Technologies, Carlsbad, CA, USA).

### ATP-binding assays

Primers flanking the MDM4 RING domain were used to amplify RING sequences, and PCR products were cloned (or cloned and mutagenized) in the pGST-parallel2 plasmid. Glutathione *S*-transferase (GST) fusion proteins were expressed in BL21 (DE3) cells. After induction for 16 hours at 20°C with 0.2 mM IPTG (isopropyl-β-d-thiogalactopyranoside), soluble proteins were extracted by sonication in lysis buffer [50 mM tris (pH 7.0), 300 mM LiSO_4_, 1 mM dithiothreitol (DTT), 0.5 mM phenylmethylsulfonyl fluoride (PMSF), 0.2% NP-40, complete Protease inhibitors (Roche) 1×]. The soluble protein fraction was incubated with Glutathione Sepharose beads (Pharmacia) at 4°C for 2 hours, and the bound proteins were washed with 50 mM tris (pH 7.0), 300 mM LiSO_4_, and 1 mM DTT and then eluted with an elution buffer [50 mM tris-HCl (pH 7.5), 300 mM NaCl, 1 mM DTT, and 15 mM glutathione]. WT and mutant GST-RING proteins (0, 1, 2, 4, or 8 μg) or GST alone (0 or 8 μg) was incubated with 10 nM ATP and 5 μCi ATP-γ^32^P for 10 min at room temperature, filtered through nitrocellulose, and counted by liquid scintillation. Alternatively, 7 μg of either WT or mutant GST-RING proteins was incubated with 5 μCi ATP-γ^32^P for 10 min at room temperature and increasing amounts (0, 0.02, 2, 20, and 200 μM) of ATP or guanosine triphosphate (GTP), filtered through nitrocellulose, and counted by liquid scintillation.

### Yeast two-hybrid assays

The yeast two-hybrid assays were performed as described (*47*). Briefly, MDM4 and MDM2 RING open reading frames were cloned in plasmids derived from the two-hybrid vectors pGADT7 (Gal4-activating domain) and pGBKT7 (Gal4-binding domain) creating N-terminal fusions and transformed in yeast haploid strains Y187 and AH109 (Clontech). Interactions were scored, after mating and diploid selection on dropout medium without leucine and tryptophan, as growth on dropout medium without leucine, tryptophan, histidine, and adenine.

### Transfection assays

U2OS cells (10^6^) were transfected by using Lipofectamine 2000 (Invitrogen) with pCDNA3.1 (6 μg), or 5 × 10^6^ cells were transfected with 30 μg of pCDNA3.1-MycTag-MDM4^WT^ or pCDNA3.1-MycTag-MDM4^TM^. Twenty-four hours after transfection, cells were treated with cycloheximide (50 μg/ml; Sigma-Aldrich, C4859), then scratched in phosphate-buffered saline (PBS) after 2, 4, or 8 hours, pelleted, and snap frozen in liquid nitrogen before protein or RNA extraction with standard protocols.

### Mdm4^T454M^ targeting construct

The targeting vector was generated by recombineering from the RP23-365M5 BAC (bacterial artificial chromosome) clone (CHORI BACPAC Resources) containing mouse *Mdm4* and downstream sequences of C57Bl6/J origin. A loxP-flanked neomycin cassette (Neo) and a diphtheria toxin α gene (DTA) were inserted downstream of the *Mdm4* gene, respectively, for positive and negative selections, and a single-nucleotide mutation encoding the missense mutation T454M (TM) was targeted in the exon 11 of *Mdm4*. The targeting construct was fully sequenced before use.

### Targeting in ES cells and mouse genotyping

CK-35 ES cells were electroporated with the targeting construct linearized with Not I. Recombinant clones were identified by long-range PCR, confirmed by Southern blot, PCR, and DNA sequencing (primer sequences in table S4). Two independent recombinant clones were injected into blastocysts to generate chimeras, and germline transmission was verified by genotyping their offspring. Reverse transcription PCR (RT-PCR) of RNAs from Mdm4^TM/TM^ MEFs showed that the mutant complementary DNA (cDNA) differed from an Mdm4 WT sequence only by the engineered missense mutation. The genotyping of *p53*^*+/−*^, *p53*^*+/*∆*31*^, and G3 *Terc^−/−^* mice was performed as previously described (*5*, *12*). All experiments were performed according to Institutional Animal Care and Use Committee regulations.

### Cells and cell culture reagents

MEFs isolated from 13.5-day embryos were cultured in a 5% CO_2_ and 3% O_2_ incubator, in Dulbecco’s modified Eagle’s medium GlutaMAX (Gibco), with 15% fetal bovine serum (Biowest), 100 μM 2-mercaptoethanol (Millipore), 0.01 mM Non-Essential Amino Acids, and penicillin/streptavidin (Gibco) for five or fewer passages, except for 3T3 experiments, performed in a 5% CO_2_ incubator for seven passages. Cells were treated for 24 hours with 10 μM Nutlin 3a (Sigma-Aldrich) (*22*) or 15 μM cisplatin (Sigma-Aldrich). Primary human fibroblasts at low passage (p.2 for *TINF2^K280E^*, p.3 for NCI-226-4 and NCI-226-8, and p.4 for *TERT^P704S^*) were thawed and cultured in fibroblast basal medium (Lonza) with 20% fetal calf serum, l-glutamin, 10 mM Hepes, penicillin/streptavidin, and gentamicin before quantitative PCR (qPCR) analysis.

### Quantitative RT-PCR

Total RNA, extracted using NucleoSpin RNA II (Macherey-Nagel), was reverse transcribed using SuperScript IV (Invitrogen), with, for TERRA quantification, a (CCCTAA)_4_ oligo as described (*48*). Real-time qPCRs were performed with primer sequences as described (*5*, *9*, *48*) on a QuantStudio using Power SYBR Green (Applied Biosystems).

### Western blots

Protein detection by immunoblotting was performed using antibodies against Mdm2 (4B2), Mdm4 (M0445; Sigma-Aldrich), p53 (AF1355, R&D Systems), actin (A2066; Sigma-Aldrich), p21 (F5; Santa Cruz Biotechnology), Myc-Tag (SAB2702192; Sigma-Aldrich), and Rtel1 (from J.-A.L.-V.). Chemiluminescence revelation was achieved with SuperSignal West Dura (Perbio). Bands of interest were quantified by using ImageJ and normalized with actin.

### Telomeric quantitative FISH

Cells were treated with colcemide (0.5 μg/ml) for 1.5 hours, submitted to hypotonic shock, fixed in an (3:1) ethanol/acetic acid solution, and dropped onto glass slides. Quantitative FISH was then carried out as described (*5*) with a TelC-Cy3 peptide nucleic acid (PNA) probe (Panagene). Images were acquired using a Zeiss Axioplan 2, and telomeric signals were quantified with iVision (Chromaphor).

### Telomeric flow-FISH

Flow-FISH with mouse cells was performed as described (*45*). For each animal, either the lungs were collected or the bone marrow from two tibias and two femurs was collected and red blood cells were lysed; then, 2 × 10^6^ cells were fixed in 500 μl of PNA hybridization buffer [70% deionized formamide, 20 mM tris (pH 7.4), and 0.1% Blocking reagent; Roche] and stored at −20°C. Either nothing (control) or 5 μl of probe stock solution was added to cells [probe stock solution: 10 μM TelC-FAM PNA probe (Panagene), 70% formamide, and 20 mM tris (pH 7.4)], and samples were denatured for 10 min at 80°C before hybridization for 2 hours at room temperature. After three washes, cells were resuspended in PBS 1×, 0.1% bovine serum albumin, ribonuclease A (1000 U/ml), and propidium iodide (12.5 μg/ml) and analyzed with an LSR II fluorescence-activated cell sorter. WT and G3 *Terc^−/−^* mice were included in all flow-FISH experiments, respectively, as controls of normal and short telomeres. For fluorescence shift analyses, the green histograms (corresponding to cells with the telomeric probe) were sliced into 18 windows of equal width and numbered 0 to 17 according to their distance from the median value in cells without the probe, and the number of cells in each window was quantified with ImageJ. The data from two to five mice per genotype were typically used to calculate mean telomere lengths, expressed relative to the mean in WT cells.

### Anatomopathology and hemograms

Organs were fixed in formol 4% for 24 hours and then ethanol 70% and embedded in paraffin wax. Serial sections were stained with hematoxylin and eosin using standard procedures (*49*). For hemograms, 100 μl of blood from each animal was recovered retro-orbitally in a 10-μl citrate-concentrated solution (S5770; Sigma-Aldrich) and analyzed using an MS9 machine (Melet Schloesing Laboratoires).

### Human polymorphisms affecting the p53 pathway

DNA extracted from Epstein-Barr virus–transformed lymphocytes of NCI-226 family members was amplified with primers flanking nucleotide polymorphisms of interest (primer sequences in table S5), and then PCR products were analyzed by Sanger DNA sequencing.

### Statistical analyses

Analyses with Student’s *t*, Mann-Whitney, or Mantel-Cox statistical tests were performed by using GraphPad Prism, and values of *P* < 0.05 were considered significant.

## Supplementary Material

aay3511_SM.pdf

## SUPPLEMENTARY MATERIALS

Supplementary material for this article is available at http://advances.sciencemag.org/cgi/content/full/6/15/eaay3511/DC1

## Appendix

View/request a protocol for this paper from *Bio-protocol*.
