# Supplementary material for: Germline mutation of MDM4, a major p53 regulator, in a familial syndrome of defective telomere maintenance
Source: Sci Adv. 2020 Apr 10;6(15):eaay3511. doi: 10.1126/sciadv.aay3511 (PMC7148086; doi:10.1126/sciadv.aay3511)
Supplement: aay3511_SM.pdf [file aay3511_SM.pdf]

Science Advances
AAAS

advances.sciencemag.org/cgi/content/full/6/15/eaay3511/DC1

Supplementary Materials for

Germline mutation of *MDM4*, a major p53 regulator, in a familial syndrome of defective telomere maintenance

Eléonore Toufektchan, Vincent Lejour, Romane Durand, Neelam Giri, Irena Draskovic, Boris Bardot, Pierre Laplante, Sara Jaber, Blanche P. Alter, José-Arturo Londono-Vallejo, Sharon A. Savage, Franck Toledo*

*Corresponding author. Email: franck.toledo@curie.fr

Published 10 April 2020, *Sci. Adv.* **6**, eaay3511 (2020)
DOI: 10.1126/sciadv.aay3511

This PDF file includes:

Figs. S1 to S5
Table S1 to S5
References

Supplementary Materials

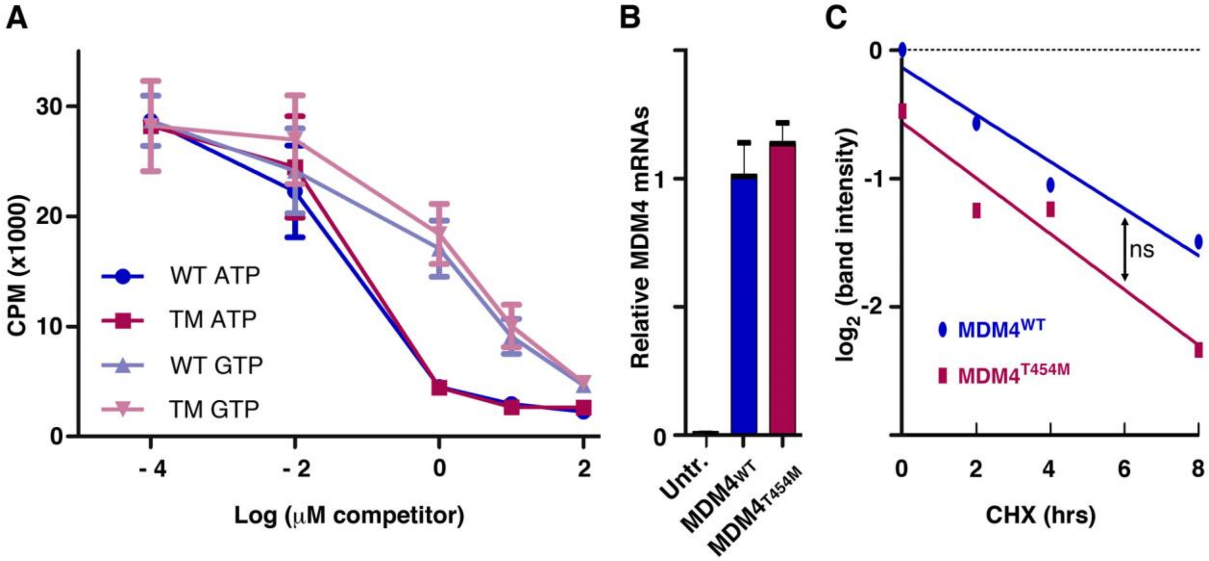

Fig. S1. Impact of the mutation on MDM4 function. (A) The WT and TM mutant RING domains bind ATP, rather than GTP. 7 μg of either WT or mutant GST-RING proteins were incubated with 5 μCi ATP-γ^{32}P for 10 min at 30°C and increasing amounts (0, 0.02, 2, 20, 200 μM) of ATP or GTP, filtered through nitrocellulose, and counted by liquid scintillation. Results from 2 independent experiments. **(B)** U2OS cells were transfected with the Myc-MDM4^{WT} or Myc-MDM4^{T454M} plasmids at similar efficiencies. mRNAs were extracted from untransfected (Untr.) U2OS cells, or cells transfected with a Myc-MDM4^{WT} or Myc-MDM4^{T454M} expression plasmid, and MDM4 mRNAs were quantified using real-time PCR, normalized to control mRNAs, then the value in cells transfected with the Myc-MDM4^{WT} plasmid was assigned a value of 1. The same transfection experiments were used for RNA extractions in this Figure and protein extractions in Fig. 1G, so that the experiments are directly comparable. Importantly, because endogenous MDM4 mRNAs were in negligible amounts compared to Myc-MDM4 mRNAs expressed from transfected plasmids, the effects on p21 levels of Myc-MDM4^{WT} and Myc-MDM4^{T454M} were directly measurable in Fig. 1G. **(C)** In transfected cells, the Myc-MDM4^{WT} and Myc-MDM4^{T454M} proteins are expressed at different levels, but exhibit similar half-lives. For half-life determination, the Myc-MDM4 bands shown in Fig. 1G were quantified and normalized to actin bands, then to the first time-point (CHX 0) of cells transfected with the Myc-MDM4^{WT} plasmid. The comparative analysis of regression lines with Graphpad prism indicated that slopes were not significantly different (ns), indicating similar protein stability.

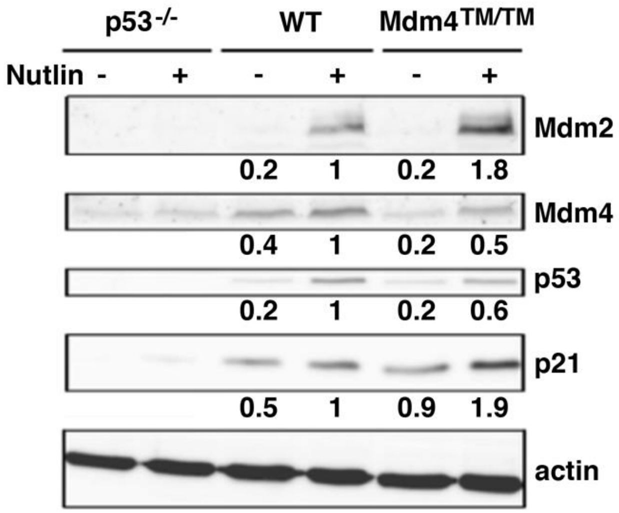

Fig. S2. Impact of the Mdm4^{T454M} mutation on p21 and Mdm2 protein levels. Protein extracts, prepared from *p53$^{-/-}$*, WT, and *Mdm4$^{TM/TM}$* MEFs before or after treatment for 24 hours with 10 μM Nutlin, were immunoblotted with antibodies against Mdm2, Mdm4, p53, p21 or actin. Bands were normalized to actin, then the values in Nutlin-treated WT MEFs were assigned a value of 1. This figure presents an experiment independent from the one presented in Fig. 3B. In the mutant cells, increased Mdm2 and p21 were observed in all (> 3) experiments (e.g. see also Fig. 3B), indicating increased p53 activity. The decreased Mdm4 protein levels in *Mdm4$^{TM/TM}$* MEFs were also observed in all experiments (see also Fig. 2G and 3B). For p53 levels however, we observed either a slight increase (as in Fig. 3B), or a slight decrease (as shown here) in the mutant cells, depending on the experiment. We presume that in some experiments the increased Mdm2 proteins are not fully inhibited by Nutlin, leading to increased p53 ubiquitination and degradation in the mutant cells. This is reminiscent of what was previously observed in another mouse model, in which Mdm4 loss was found to increase p53 activity, but decrease p53 stability (*53*).

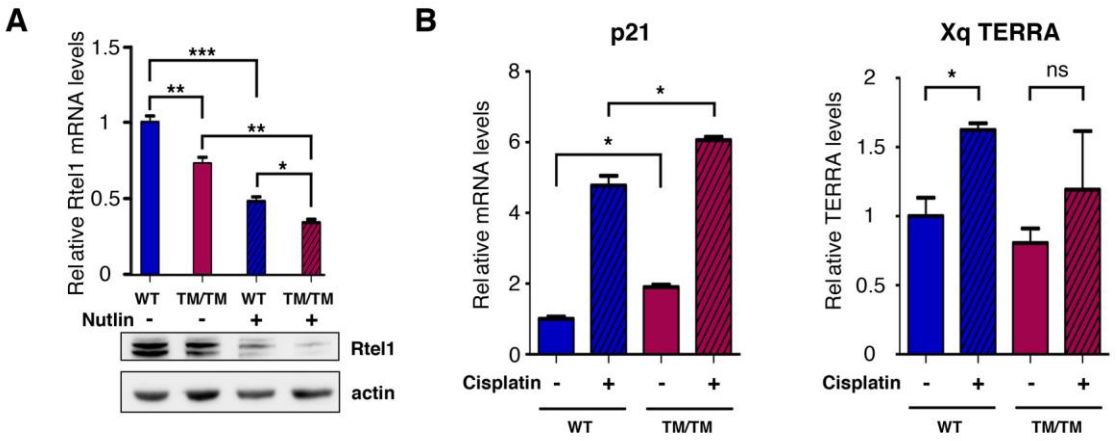

Fig. S3. Quantification of Rtel1 and TERRA in MEFs. **(A)** Quantification of Rtel1 mRNAs extracted from WT and *Mdm4*$^{TM/TM}$ MEFs, unstressed or Nutlin-treated. Results from 3 experiments. Below, western blots showing Rtel1 and actin protein levels. **(B)** Quantification of p21 and TERRA extracted from WT and *Mdm4*$^{TM/TM}$ MEFs, unstressed or treated with 15 μM cisplatin. Results from 2 independent MEFs per genotype.

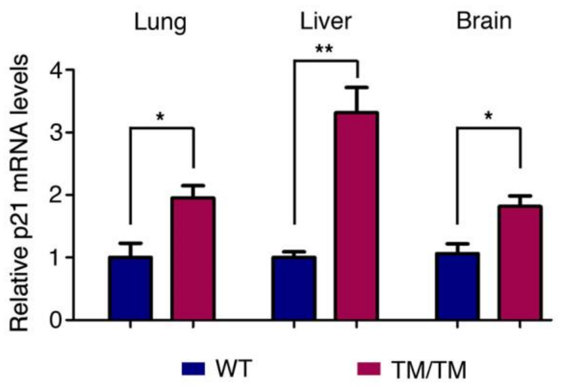

Fig. S4. Tissues from *Mdm4*$^{TM/TM}$ pups exhibit increased p21 mRNA levels. mRNAs were extracted from the lungs, liver and brain of WT and *Mdm4*$^{TM//TM}$ P0 pups, then p21 mRNA levels were quantified using real-time PCR, normalized to control mRNAs, and the value in WT tissues was assigned a value of 1. Results from 3 WT and 4 *Mdm4*$^{TM//TM}$ pups. * $P<0.05$ and ** $P<0.01$ by Student's t tests.

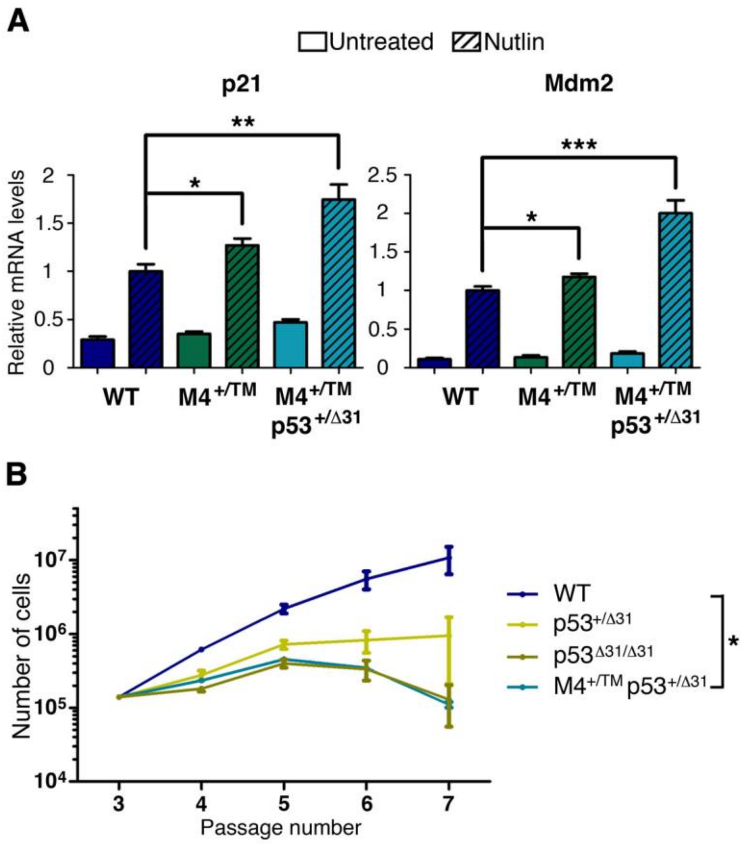

Fig. S5. *Mdm4*$^{+/TM}$ *p53*$^{+/\Delta 31}$ MEFs exhibit increased p53 activity. (A) Quantification of p21 and Mdm2 mRNAs extracted from WT, *Mdm4*$^{+/TM}$ (M4$^{+/TM}$) and *Mdm4*$^{+/TM}$ *p53*$^{+/\Delta 31}$ (M4$^{+/TM}$ p53$^{+/\Delta 31}$) MEFs, treated or not 10 μM Nutlin for 24 hours. p21 and Mdm2 mRNAs were quantified as in Fig. 3A. Results from 4 independent experiments, and at least 2 independent MEFs per genotype. * $P<0.05$, ** $P<0.01$ and *** $P<0.001$ by Student's t tests. **(B)** Proliferation in a 3T3 protocol of MEFs of the indicated genotypes. Each point is the average value of 2 independent MEFs. * $P<0.05$ by Student's t test at passage 7.

NCI ID	Gene, variant	Hematologic manifestations	Other clinical manifestations
226-1, proband	*MDM4* p.T454M<br><br>*TERT* p.W203S	Current age 29 years.<br>Mild intermittent neutropenia from age 14 years (ANC range 1.0-1.5 x 10^9/L)<br>Macrocytosis: MCV 100-105 (normal 79-92.2 fL)<br>Hb 14.4 gm/dL, platelets 144 x 10^9/L<br>EPO 77.3 (normal 3.7-31.5 mIU/mL)<br>HbF 8.5% (normal 0-2%)<br>Bone marrow hypocellular (30%), no dysplasia. Cytogenetics 46,XY[20]	No dysmorphology; no DC phenotype; normal weight and height.<br>Chronic fatigue and muscle pains since age 14, currently being treated with gabapentin.<br>Normal PFT, MRI brain, LFT
226-2, sister	*MDM4* p.T454M	Current age 31 years.<br>WBC range 3.6 – 4.1 x 10^9/L, ANC 2.5 x 10^9/L, Hb 13.2 gm/dL, platelets 193 x 10^9/L; MCV 95.2 (normal 79.4-94.8 fL).<br>Mild lymphopenia with ALC 0.85 x 10^9/L (normal 1.18-3.74 x 10^9/L)<br>EPO 27.6 (normal 3.7-31.5 mIU/mL)<br>HbF <1% (normal 0-2%)	No dysmorphology; no DC phenotype; normal weight and height.<br>Recurrent sinus and respiratory infections needing treatment with prolonged antibiotic courses. B cell immunodeficiency with poor response to vaccines. Chronic fatigue and muscle pains being treated with gabapentin
226-3, father	*TERT* p.W203S	CBC normal at age 47 years. MCV 89 fL<br>Hemochromatosis mutation positive (*HFE* p.C282Y/H63D)	No abnormalities
226-4, mother	*MDM4* p.T454M	Current age 55 years.<br>Macrocytosis: MCV 100-107 (normal 79.4-94.8 fL). WBC range 2.6 – 4.0 x 10^9/L, ANC 1.7 x 10^9/L, Hb 14.2 gm/dL, platelets 209 x 10^9/L.<br>EPO 64.4 (normal 3.7-31.5 mIU/mL)<br>HbF 4.1% (normal 0-2%)<br>Bone marrow hypocellular (20-30%); no dysplasia; 46,XX[20]	No dysmorphology; no DC phenotype; normal weight and height.<br>H/O menorrhagia; thin hair; hypothyroidism; osteopenia<br>Normal PFT; normal LFT
226-7, aunt	*MDM4* p.T454M	Died at age 52 from acute myeloid leukemia	History of melanoma
226-8, cousin	*MDM4* p.T454M	Current age 41 years.<br>Macrocytosis: MCV 100-108 (normal 79.4-94.8 fL). ANC 1.7 x 10^9/L; Hb 14.8 gm/dL, platelets 217 x 10^9/L.<br>EPO 22.6, (normal 3.7-31.5 mIU/mL)<br>HbF <1% (normal 0-2%)<br>Bone marrow: hypocellular (30-50%), no dysplasia. Cytogenetics: 46,XX[20]	No dysmorphology; no DC phenotype; normal weight and height.<br>Tongue SCC resected at age 27 years.<br>Fertility problems and h/o fetal losses.<br>Received fertility meds; preeclampsia, preterm delivery
226-9, cousin	DNA not available	None known	HNSCC at 42 years, died of cancer-related complications

Table S1. Clinical data on study participants. Participants were evaluated by the study team and data reflect summary of eleven years of follow-up for each participant. DNA from the proband's maternal aunt (226-7) was not available, but she is an obligate carrier given the genotypes of her sister and daughter. Abbreviations: ANC, absolute neutrophil count; MCV, mean corpuscular volume; Hb, hemoglobin; EPO, erythropoietin; HbF, hemoglobin F; DC phenotype, dyskeratosis congenita mucocutaneous triad; WBC, white blood cells; ALC, absolute lymphocyte count; CBC, complete blood count; PFT, pulmonary function tests; LFT, liver function tests; SCC, squamous cell carcinoma; HNSCC, head and neck squamous cell carcinoma.

Chromosome	Position	ID	REF	ALT	gnomAD allele count	gnomAD MAF^	Ensembl GeneName	Ensembl Transcript	Amino Acid Change	REVEL Score	CADD_phred Score	MetaSVM	Proband, NCI-226-1	Sister, NCI226-2	Father, NCI-226-3	Mother, NCI-226-4	Maternal Cousin, NCI-226-8
chr1	147084719	rs144335253*	C	G	5/279938	0.00001786	*BCL9*	ENST00000234739	p.Pro31Ala	0.166	27.1	T	0/1	0/0	0/0	0/1	0/1
chr1	204518698	.	C	T	0	0	*MDM4*	ENST00000367182	p.Thr454Met	0.872	28	D	0/1	0/1	0/0	0/1	0/1
chr1	205273017	.	G	C	0	0	*NUAK2*	ENST00000367157	p.Pro483Arg	0.088	10.6	T	0/1	0/1	0/0	0/1	0/1
chr2	109067508	rs148209837	C	A	135/282670	0.0004776	*GCC2*	ENST00000309863	p.Leu30Ile	0.198	21.2	T	0/1	0/1	0/0	0/1	0/1
chr2	220162145	rs151265781	C	T	16/282100	0.00005672	*PTPRN*	ENST00000295718	p.Arg633His	0.38	22.4	T	0/1	0/0	0/0	0/1	0/1
chr7	33945314	rs367821838	A	G	7/250366	0.00002796	*BMPER*	ENST00000297161	p.Asn30Ser	0.078	18.65	T	0/1	0/1	0/0	0/1	0/1
chr9	103109174	rs1235691348	C	T	2/251420	0.000007955	*TEX10*	ENST00000374902	p.Ser232Asn	0.066	17.53	T	0/1	0/0	0/0	0/1	0/1
chr9	115336621	rs375495090	A	G	2/282506	0.000007079	*KIAA1958*	ENST00000536272	p.Ile87Met	0.27	0.367	T	0/1	0/0	0/0	0/1	0/1
chr10	104125263	rs1373756831	A	G	2/282370	0.00001062	*GBF1*	ENST00000369983	p.Gln738Arg	0.047	21	T	0/1	0/0	0/0	0/1	0/1
chr11	66055605	.	T	C	0	0	*YIF1A*	ENST00000376901	p.Met64Val	0.149	22.3	T	0/1	0/1	0/0	0/1	0/1
chr12	53164963	rs199792968	C	T	14/282872	0.00004949	*KRT76*	ENST00000332411	p.Arg435His	0.609	25.2	D	0/1	0/0	0/0	0/1	0/1
chr13	49281319	rs201226412	G	A	2/282452	0.000007081	*CYSLTR2*	ENST00000282018	p.Met122Ile	0.45	27.4	T	0/1	0/1	0/0	0/1	0/1
chr13	111562966	rs143331283	G	A	7/250770	0.00002791	*ANKRD10*	ENST00000267339	p.Pro121Leu	0.091	23.2	T	0/1	0/1	0/0	0/1	0/1
chr14	86087877	rs747657938	A	G	3/249689	0.00001202	*FLRT2*	ENST00000330753	p.Lys7Glu	0.063	19.81	T	0/1	0/0	0/0	0/1	0/1
chr17	78078662	rs142481170	G	A	22/279638	0.00007867	*GAA*	ENST00000302262	p.Ala93Thr	0.284	15.61	T	0/1	0/1	0/0	0/1	0/1
chr20	30070192	rs200495484	C	T	20/251304	0.00007958	*REM1*	ENST00000201979	p.Arg176Cys	0.862	22.5	D	0/1	0/0	0/0	0/1	0/1

Table S2. Variants identified in exome sequencing of family NCI-226 as described in the Methods.

^Minor allele frequency (MAF) reported for all gnomAD populations combined (*52*).

*rs144335253 is reported as multi-allelic C>G,T. The T allele is absent in gnomAD.

REVEL score determined by Ioannidis *et al.* (*54*).

Phenotype	Mdm4 mutants	compound mutants	p53 mutants
Embryonic lethality	$Mdm4^{-/-}$		
Perinatal lethality	$Mdm4^{TM/TM}$		
Bone Marrow failure<br>(65-100% of mice in < 5 months)		$Mdm4^{+/TM}$ $p53^{+/\Delta31}$<br>$Mdm4^{TM/TM}$ $p53^{+/-}$	$p53^{\Delta31/\Delta31}$
Rare accelerated death<br>(2-6% of mice in < 7 months)	$Mdm4^{+/TM}$		$p53^{+/\Delta31}$
WT	$Mdm4^{+/+}$		$p53^{+/+}$
Cancer after > 1 year			$p53^{+/-}$
Cancer in < 1 year		$Mdm4^{TM/TM}$ $p53^{-/-}$	$p53^{-/-}$

Table S3. A summary of phenotypes of Mdm4 and p53 mutants. Background colors indicate p53 activity, from null (white) to very high (black). The phenotypes of $Mdm4^{-/-}$, $p53^{-/-}$, $p53^{+/-}$, $p53^{+/\Delta31}$ and $p53^{\Delta31/\Delta31}$ mice were described previously (*5*,*55*,*56*), whereas those of $Mdm4^{+/TM}$, $Mdm4^{TM/TM}$ and compound mutants are described in this report.

Primer Name	Purpose	Sequence 5’-3’
a	PCR screen (3’) of ES cells	TAGGTCCCTCGAGGGGATC
b	PCR screen (3’) of ES cells	CGTAGGCAATTCTGTAACCACAAC
c	Amplification of mutated region	ACTCAGTGGGATTTTTGGATTTGG
d	Amplification of mutated region	CCCTCTGGGTTCACAATTTGG
e	ES and mouse genotyping	GTTTGCCGAGGAACTTTCCT
f	ES and mouse genotyping	GCAAGACTCTGCCCCAAAAG
SB-5’-F	PCR product is 5’ Southern blot probe	CAGCCTTCTGTAAGGCAGC
SB-5’-R	PCR product is 5’ Southern blot probe	TAAAAACCACAGGGCTACTAAATACC
SB-3’-F	PCR product is 3’ Southern blot probe	GACTGCCTTGAAGATGGAGGAA
SB-3’-R	PCR product is 3’ Southern blot probe	CACTTCCCCAAATCATGCCAAA
SeqTM	Sequencing of mutation	CATAGTTCTGAAAGCCAGGAGA

Table S4. Primers for the targeting strategy and mouse genotyping. These primers are relevant to Figure 2.

Primer Name	Tested polymorphisms	Sequence 5’-3’
SNPp53F	rs1800371, rs1042522, rs17878362	TGGGACTGACTTTCTGCTCT
SNPp53R	rs1800371, rs1042522, rs17878362	CAAGAAGCCCAGACGGAAAC
p53-2F	rs17880560	ATGTCTGACTGCCCCTTCAA
p53-2R	rs17880560	CTGCATGACAACAAGGGAGG
p53-2S	rs17880560	TCCCTATCCCAAGAAGCCCT
SNPm2F	rs117039649, rs2279744	GCTTTGCGGAGGTTTTGTTGGACT
SNPm2R	rs117039649, rs2279744	CCACAGGTCTACCCTCCAATCGCCACT
SNPm4-1F	rs4245739	GCCAAGAGACCATCTCAAGC
SNPm4-1R	rs4245739	ACTGTCTGAAGAGGGGCAGA
SNPm4-2F	rs11801299	CCTAGCTCAGCCTCCCAAAA
SNPm4-2R	rs11801299	TCCCAGCAGCCATACTTCAA
SNPm4-2S	rs11801299	TTGCTTTTGCCTGGCACAAT
SNPm4-3F	rs1380576	CTTCTGGCCTGGAATCTCGT
SNPm4-3R	rs1380576	TTACAGGCATGCACCATCAC

Table S5. Primers for human polymorphism genotyping. The primers were used to amplify and genotype the variants mentioned in Fig. 6A.

REFERENCES AND NOTES

1. J. M. Nigro, S. J. Baker, A. C. Preisinger, J. M. Jessup, R. Hosteller, K. Cleary, S. H. Signer, N. Davidson, S. Baylin, P. Devilee, T. Glover, F. S. Collins, A. Weslon, R. Modali, C. C. Harris, B. Vogelstein, Mutations in the p53 gene occur in diverse human tumour types. *Nature* **342**, 705–708 (1989).

2. D. Malkin, F. P. Li, L. C. Strong, J. F. Fraumeni, C. E. Nelson, D. H. Kim, J. Kassel, M. A. Gryka, F. Z. Bischoff, M. A. Tainsky, Germ line p53 mutations in a familial syndrome of breast cancer, sarcomas, and other neoplasms. *Science* **250**, 1233–1238 (1990).

3. G. L. Bond, W. Hu, E. E. Bond, H. Robins, S. G. Lutzker, N. C. Arva, J. Bargonetti, F. Bartel, H. Taubert, P. Wuerl, K. Onel, L. Yip, S.-J. Hwang, L. C. Strong, G. Lozano, A. J. Levine, A single nucleotide polymorphism in the MDM2 promoter attenuates the p53 tumor suppressor pathway and accelerates tumor formation in humans. *Cell* **119**, 591–602 (2004).

4. J. Wynendaele, A. Böhnke, E. Leucci, S. J. Nielsen, I. Lambertz, S. Hammer, N. Sbrzesny, D. Kubitza, A. Wolf, E. Gradhand, K. Balschun, I. Braicu, J. Sehouli, S. Darb-Esfahani, C. Denkert, C. Thomssen, S. Hauptmann, A. Lund, J.-C. Marine, F. Bartel, An illegitimate microRNA target site within the 3' UTR of MDM4 affects ovarian cancer progression and chemosensitivity. *Cancer Res.* **70**, 9641–9649 (2010).

5. I. Simeonova, S. Jaber, I. Draskovic, B. Bardot, M. Fang, R. Bouarich-Bourimi, V. Lejour, L. Charbonnier, C. Soudais, J.-C. Bourdon, M. Huerre, A. Londono-Vallejo, F. Toledo, Mutant mice lacking the p53 C-terminal domain model telomere syndromes. *Cell Rep.* **3**, 2046–2058 (2013).

6. S. A. Savage, Beginning at the ends: Telomeres and human disease. *F1000Res.* **7**, 524 (2018).

7. B. P. Alter, N. Giri, S. A. Savage, P. S. Rosenberg, Cancer in the National Cancer Institute inherited bone marrow failure syndrome cohort after fifteen years of follow-up. *Haematologica* **103**, 30–39 (2018).

8. S. C. Ward, S. A. Savage, N. Giri, B. P. Alter, P. S. Rosenberg, D. C. Pichard, E. W. Cowen, Beyond the triad: Inheritance, mucocutaneous phenotype, and mortality in a cohort of patients with dyskeratosis congenita. *J. Am. Acad. Dermatol.* **78**, 804–806 (2018).

9. S. Jaber, E. Toufektchan, V. Lejour, B. Bardot, F. Toledo, p53 downregulates the Fanconi anaemia DNA repair pathway. *Nat. Commun.* **7**, 11091 (2016).

10. D. Filipescu, M. Naughtin, K. Podsypanina, V. Lejour, L. Wilson, Z. A. Gurard-Levin, G. A. Orsi, I. Simeonova, E. Toufektchan, L. D. Attardi, F. Toledo, G. Almouzni, Essential role for centromeric factors following p53 loss and oncogenic transformation. *Genes Dev.* **31**, 463–480 (2017).

11. E. Toufektchan, F. Toledo, The guardian of the genome revisited: p53 downregulates genes required for telomere maintenance, DNA repair, and centromere structure. *Cancer* **10**, E135 (2018).

12. M. A. Blasco, H. W. Lee, M. P. Hande, E. Samper, P. M. Lansdorp, R. A. DePinho, C. W. Greider, Telomere shortening and tumor formation by mouse cells lacking telomerase RNA. *Cell* **91**, 25–34 (1997).

13. E. Aix, Ó. Gutiérrez-Gutiérrez, C. Sánchez-Ferrer, T. Aguado, I. Flores, Postnatal telomere dysfunction induces cardiomyocyte cell-cycle arrest through p21 activation. *J. Cell Biol.* **213**, 571–583 (2016).

14. D. Hockemeyer, W. Palm, R. C. Wang, S. S. Couto, T. de Lange, Engineered telomere degradation models dyskeratosis congenita. *Genes Dev.* **22**, 1773–1785 (2008).

15. S. Kim, J.-H. Jhong, J. Lee, J.-Y. Koo, Meta-analytic support vector machine for integrating multiple omics data. *BioData Min.* **10**, 2 (2017).

16. C. Priest, C. Prives, M. V. Poyurovsky, Deconstructing nucleotide binding activity of the Mdm2 RING domain. *Nucleic Acids Res.* **38**, 7587–7598 (2010).

17. K. Linke, P. D. Mace, C. A. Smith, D. L. Vaux, J. Silke, C. L. Day, Structure of the MDM2/MDMX RING domain heterodimer reveals dimerization is required for their ubiquitylation in trans. *Cell Death Differ.* **15**, 841–848 (2008).

18. J. E. Landers, S. L. Cassel, D. L. George, Translational enhancement of mdm2 oncogene expression in human tumor cells containing a stabilized wild-type p53 protein. *Cancer Res.* **57**, 3562–3568 (1997).

19. B. X. Tan, H. P. Liew, J. S. Chua, F. J. Ghadessy, Y. S. Tan, D. P. Lane, C. R. Coffill, Anatomy of Mdm2 and Mdm4 in evolution. *J. Mol. Cell Biol.* **9**, 3–15 (2017).

20. B. Bardot, R. Bouarich-Bourimi, J. Leemput, V. Lejour, A. Hamon, L. Plancke, A. G. Jochemsen, I. Simeonova, M. Fang, F. Toledo, Mice engineered for an obligatory Mdm4 exon skipping express higher levels of the Mdm4-S isoform but exhibit increased p53 activity. *Oncogene* **34**, 2943–2948 (2015).

21. M. Dewaele, T. Tabaglio, K. Willekens, M. Bezzi, S. X. Teo, D. H. P. Low, C. M. Koh, F. Rambow, M. Fiers, A. Rogiers, E. Radaelli, M. Al-Haddawi, S. Y. Tan, E. Hermans, F. Amant, H. Yan, M. Lakshmanan, R. C. Koumar, S. T. Lim, F. A. Derheimer, R. M. Campbell, Z. Bonday, V. Tergaonkar, M. Shackleton, C. Blattner, J.-C. Marine, E. Guccione, Antisense oligonucleotide-mediated MDM4 exon 6 skipping impairs tumor growth. *J. Clin. Invest.* **126**, 68–84 (2016).

22. L. T. Vassilev, B. T. Vu, B. Graves, D. Carvajal, F. Podlaski, Z. Filipovic, N. Kong, U. Kammlott, C. Lukacs, C. Klein, N. Fotouhi, E. A. Liu, In vivo activation of the p53 pathway by small-molecule antagonists of MDM2. *Science* **303**, 844–848 (2004).

23. B. Hu, D. M. Gilkes, B. Farooqi, S. M. Sebti, J. Chen, MDMX overexpression prevents p53 activation by the MDM2 inhibitor Nutlin. *J. Biol. Chem.* **281**, 33030–33035 (2006).

24. M. Wade, E. T. Wong, M. Tang, J. M. Stommel, G. M. Wahl, Hdmx modulates the outcome of p53 activation in human tumor cells. *J. Biol. Chem.* **281**, 33036–33044 (2006).

25. S. Tutton, G. A. Azzam, N. Stong, O. Vladimirova, A. Wiedmer, J. A. Monteith, K. Beishline, Z. Wang, Z. Deng, H. Riethman, S. B. McMahon, M. Murphy, P. M. Lieberman, Subtelomeric p53 binding prevents accumulation of DNA damage at human telomeres. *EMBO J.* **35**, 193–207 (2016).

26. S. Tutton, Z. Deng, N. Gulve, O. Vladimirova, K. Beishline, A. Wiedmer, M. Murphy, P. M. Lieberman, Elevated telomere dysfunction in cells containing the African-centric Pro47Ser cancer-risk variant of TP53. *Oncotarget* **10**, 3581–3591 (2019).

27. F. Toledo, p53: A two-faced regulator of telomere metabolism? (comment on DOI 10.1002/bies.201600078). *Bioessays* **38**, 938 (2016).

28. M. C. Marin, C. A. Jost, L. A. Brooks, M. S. Irwin, J. O'Nions, J. A. Tidy, N. James, J. M. McGregor, C. A. Harwood, I. G. Yulug, K. H. Vousden, M. J. Allday, B. Gusterson, S. Ikawa, P. W. Hinds, T. Crook, W. G. Kaelin, A common polymorphism acts as an intragenic modifier of mutant p53 behaviour. *Nat. Genet.* **25**, 47–54 (2000).

29. X. Li, P. Dumont, A. Della Pietra, C. Shetler, M. E. Murphy, The codon 47 polymorphism in p53 is functionally significant. *J. Biol. Chem.* **280**, 24245–24251 (2005).

30. C. Sagne, V. Marcel, M. Bota, G. Martel-Planche, A. Nobrega, E. I. Palmero, L. Perriaud, M. Boniol, S. Vagner, D. G. Cox, C. S. Chan, J.-L. Mergny, M. Olivier, P. Ashton-Prolla, J. Hall, P. Hainaut, M. I. Achatz, Age at cancer onset in germline TP53 mutation carriers: Association with polymorphisms in predicted G-quadruplex structures. *Carcinogenesis* **35**, 807–815 (2014).

31. S. Knappskog, M. Bjørnslett, L. M. Myklebust, P. E. A. Huijts, M. P. Vreeswijk, H. Edvardsen, Y. Guo, X. Zhang, M. Yang, S. K. Ylisaukko-Oja, P. Alhopuro, J. Arola, R. A. E. M. Tollenaar, C. J. van Asperen, C. Seynaeve, V. Staalesen, R. Chrisanthar, E. Løkkevik, H. B. Salvesen, D. G. Evans, W. G. Newman, D. Lin, L. A. Aaltonen, A.-L. Børresen-Dale, G. S. Tell, C. Stoltenberg, P. Romundstad, K. Hveem, J. R. Lillehaug, L. Vatten, P. Devilee, A. Dørum, P. E. Lønning, The MDM2 promoter SNP285C/309G haplotype diminishes Sp1 transcription factor binding and reduces risk for breast and ovarian cancer in Caucasians. *Cancer Cell.* **19**, 273–282 (2011).

32. F. Yu, Z. Jiang, A. Song, Association of rs11801299 and rs1380576 polymorphisms at MDM4 with risk, clinicopathological features and prognosis in patients with retinoblastoma. *Cancer Epidemiol.* **58**, 153–159 (2019).

33. J. B. Kodal, S. Vedel-Krogh, C. J. Kobylecki, B. G. Nordestgaard, S. E. Bojesen, TP53 Arg72Pro, mortality after cancer, and all-cause mortality in 105,200 individuals. *Sci. Rep.* **7**, 336 (2017).

34. H. Tummala, A. Walne, L. Collopy, S. Cardoso, J. de la Fuente, S. Lawson, J. Powell, N. Cooper, A. Foster, S. Mohammed, V. Plagnol, T. Vulliamy, I. Dokal, Poly(A)-specific ribonuclease deficiency impacts telomere biology and causes dyskeratosis congenita. *J. Clin. Invest.* **125**, 2151–2160 (2015).

35. E. Devany, X. Zhang, J. Y. Park, B. Tian, F. E. Kleiman, Positive and negative feedback loops in the p53 and mRNA 3′ processing pathways. *Proc. Natl. Acad. Sci. U.S.A.* **110**, 3351–3356 (2013).

36. A. Son, J.-E. Park, V. N. Kim, PARN and TOE1 Constitute a 3′ end maturation module for nuclear non-coding RNAs. *Cell Rep.* **23**, 888–898 (2018).

37. S. Shukla, G. A. Bjerke, D. Muhlrad, R. Yi, R. Parker, The RNase PARN controls the levels of specific miRNAs that contribute to p53 regulation. *Mol. Cell* **73**, 1204–1216.e4 (2019).

38. D. H. Moon, M. Segal, B. Boyraz, E. Guinan, I. Hofmann, P. Cahan, A. K. Tai, S. Agarwal, Poly(A)-specific ribonuclease (PARN) mediates 3′-end maturation of the telomerase RNA component. *Nat. Genet.* **47**, 1482–1488 (2015).

39. B. Boyraz, D. H. Moon, M. Segal, M. Z. Muosieyiri, A. Aykanat, A. K. Tai, P. Cahan, S. Agarwal, Posttranscriptional manipulation of TERC reverses molecular hallmarks of telomere disease. *J. Clin. Invest.* **126**, 3377–3382 (2016).

40. D. Lessel, D. Wu, C. Trujillo, T. Ramezani, I. Lessel, M. K. Alwasiyah, B. Saha, F. M. Hisama, K. Rading, I. Goebel, P. Schütz, G. Speit, J. Högel, H. Thiele, G. Nürnberg, P. Nürnberg, M. Hammerschmidt, Y. Zhu, D. R. Tong, C. Katz, G. M. Martin, J. Oshima, C. Prives, C. Kubisch, Dysfunction of the MDM2/p53 axis is linked to premature aging. *J. Clin. Invest.* **127**, 3598–3608 (2017).

41. C. López-Otín, M. A. Blasco, L. Partridge, M. Serrano, G. Kroemer, The hallmarks of aging. *Cell* **153**, 1194–1217 (2013).

42. T. Toki, K. Yoshida, R. Wang, S. Nakamura, T. Maekawa, K. Goi, M. C. Katoh, S. Mizuno, F. Sugiyama, R. Kanezaki, T. Uechi, Y. Nakajima, Y. Sato, Y. Okuno, A. Sato-Otsubo, Y. Shiozawa, K. Kataoka, Y. Shiraishi, M. Sanada, K. Chiba, H. Tanaka, K. Terui, T. Sato, T. Kamio, H. Sakaguchi, S. Ohga, M. Kuramitsu, I. Hamaguchi, A. Ohara, H. Kanno, S. Miyano, S. Kojima, A. Ishiguro, K. Sugita, N. Kenmochi, S. Takahashi, K. Eto, S. Ogawa, E. Ito, De Novo mutations activating germline TP53 in an inherited bone-marrow-failure syndrome. *Am. J. Hum. Genet.* **103**, 440–447 (2018).

43. J. L. Van Nostrand, L. D. Attardi, Guilty as CHARGED: p53's expanding role in disease. *Cell Cycle* **13**, 3798–3807 (2014).

44. E. G. Arias-Salgado, E. Galvez, L. Planas-Cerezales, L. Pintado-Berninches, E. Vallespin, P. Martinez, J. Carrillo, L. Iarriccio, A. Ruiz-Llobet, A. Catalá, I. Badell-Serra, L. I. Gonzalez-Granado, A. Martín-Nalda, M. Martínez-Gallo, A. Galera-Miñarro, C. Rodríguez-Vigil, M. Bastos-Oreiro, G. Perez de Nanclares, V. Leiro-Fernández, M.-L. Uria, C. Diaz-Heredia, C. Valenzuela, S. Martín, B. López-Muñiz, P. Lapunzina, J. Sevilla, M. Molina-Molina, R. Perona, L. Sastre, Genetic analyses of aplastic anemia and idiopathic pulmonary fibrosis patients with short telomeres, possible implication of DNA-repair genes. *Orphanet J. Rare Dis.* **14**, 82 (2019).

45. G. M. Baerlocher, I. Vulto, G. de Jong, P. M. Lansdorp, Flow cytometry and FISH to measure the average length of telomeres (flow FISH). *Nat. Protoc.* **1**, 2365–2376 (2006).

46. B. J. Ballew, M. Yeager, K. Jacobs, N. Giri, J. Boland, L. Burdett, B. P. Alter, S. A. Savage, Germline mutations of regulator of telomere elongation helicase 1, RTEL1, in Dyskeratosis congenita. *Hum. Genet.* **132**, 473–480 (2013).

47. C. Adam, R. Guérois, A. Citarella, L. Verardi, F. Adolphe, C. Béneut, V. Sommermeyer, C. Ramus, J. Govin, Y. Couté, V. Borde, The PHD finger protein Spp1 has distinct functions in the Set1 and the meiotic DSB formation complexes. *PLOS Genet.* **14**, e1007223 (2018).

48. I. López de Silanes, O. Graña, M. L. De Bonis, O. Dominguez, D. G. Pisano, M. A. Blasco, Identification of TERRA locus unveils a telomere protection role through association to nearly all chromosomes. *Nat. Commun.* **5**, 4723 (2014).

49. E. Prophet, B. Mills, J. Arrington, L. Sobin, *Laboratory Methods in Histotechnology* (AFIP, 1992).

50. B. P. Alter, G. M. Baerlocher, S. A. Savage, S. J. Chanock, B. B. Weksler, J. P. Willner, J. A. Peters, N. Giri, P. M. Lansdorp, Very short telomere length by flow fluorescence in situ hybridization identifies patients with dyskeratosis congenita. *Blood* **110**, 1439–1447 (2007).

51. N. A. Laurie, S. L. Donovan, C.-S. Shih, J. Zhang, N. Mills, C. Fuller, A. Teunisse, S. Lam, Y. Ramos, A. Mohan, D. Johnson, M. Wilson, C. Rodriguez-Galindo, M. Quarto, S. Francoz, S. M. Mendrysa, R. K. Guy, J.-C. Marine, A. G. Jochemsen, M. A. Dyer, Inactivation of the p53 pathway in retinoblastoma. *Nature* **444**, 61–66 (2006).

52. M. Lek, K. J. Karczewski, E. V. Minikel, K. E. Samocha, E. Banks, T. Fennell, A. H. O'Donnell-Luria, J. S. Ware, A. J. Hill, B. B. Cummings, T. Tukiainen, D. P. Birnbaum, J. A. Kosmicki, L. E. Duncan, K. Estrada, F. Zhao, J. Zou, E. Pierce-Hoffman, J. Berghout, D. N. Cooper, N. Deflaux, M. DePristo, R. Do, J. Flannick, M. Fromer, L. Gauthier, J. Goldstein, N. Gupta, D. Howrigan, A. Kiezun, M. I. Kurki, A. L. Moonshine, P. Natarajan, L. Orozco, G. M. Peloso, R. Poplin, M. A. Rivas, V. Ruano-Rubio, S. A. Rose, D. M. Ruderfer, K. Shakir, P. D. Stenson, C. Stevens, B. P. Thomas, G. Tiao, M. T. Tusie-Luna, B. Weisburd, H.-H. Won, D. Yu, D. M. Altshuler, D. Ardissino, M. Boehnke, J. Danesh, S. Donnelly, R. Elosua, J. C. Florez, S. B. Gabriel, G. Getz, S. J. Glatt, C. M. Hultman, S. Kathiresan, M. Laakso, S. McCarroll, M. I. McCarthy, D. McGovern, R. McPherson, B. M. Neale, A. Palotie, S. M. Purcell, D. Saleheen, J. M. Scharf, P. Sklar, P. F. Sullivan, J. Tuomilehto, M. T. Tsuang, H. C. Watkins, J. G. Wilson, M. J. Daly, D. G. MacArthur, Exome Aggregation Consortium, Analysis of protein-coding genetic variation in 60,706 humans. *Nature* **536**, 285–291 (2016).

53. F. Toledo, K. A. Krummel, C. J. Lee, C.-W. Liu, L.-W. Rodewald, M. Tang, G. M. Wahl, A mouse p53 mutant lacking the proline-rich domain rescues Mdm4 deficiency and provides insight into the Mdm2-Mdm4-p53 regulatory network. *Cancer Cell* **9**, 273–285 (2006).

54. N. M. Ioannidis, J. H. Rothstein, V. Pejaver, S. Middha, S. K. McDonnell, S. Baheti, A. Musolf, Q. Li, E. Holzinger, D. Karyadi, L. A. Cannon-Albright, C. C. Teerlink, J. L. Stanford, W. B. Isaacs, J. Xu, K. A. Cooney, E. M. Lange, J. Schleutker, J. D. Carpten, I. J. Powell, O. Cussenot, G. Cancel-Tassin, G. G. Giles, R. J. MacInnis, C. Maier, C.-L. Hsieh, F. Wiklund, W. J. Catalona, W. D.

Foulkes, D. Mandal, R. A. Eeles, Z. Kote-Jarai, C. D. Bustamante, D. J. Schaid, T. Hastie, E. A. Ostrander, J. E. Bailey-Wilson, P. Radivojac, S. N. Thibodeau, A. S. Whittemore, W. Sieh, REVEL: An ensemble method for predicting the pathogenicity of rare missense variants. *Am. J. Hum. Genet.* **99**, 877–885 (2016).

55. L. A. Donehower, M. Harvey, B. L. Slagle, M. J. McArthur, C. A. Montgomery, J. S. Butel, A. Bradley, Mice deficient for p53 are developmentally normal but susceptible to spontaneous tumours. *Nature* **356**, 215–221 (1992).

56. J.-C. Marine, S. Francoz, M. Maetens, G. Wahl, F. Toledo, G. Lozano, Keeping p53 in check: Essential and synergistic functions of Mdm2 and Mdm4. *Cell Death Differ.* **13**, 927–934 (2006).
